# Mapping the global health burden of volcanic exposure: a scoping review approach

**DOI:** 10.3389/fpubh.2025.1658384

**Published:** 2025-09-24

**Authors:** Katherine Simbaña-Rivera, Jesús Endara-Mina, Damary S. Jaramillo-Aguilar, Leonardo D. Mera-Lojano, Ruth Jimbo-Sotomayor, Xavier Sánchez, María Cristo Rodríguez-Pérez, Manuel Enrique Fuentes-Ferrer, Luis D. Boada

**Affiliations:** ^1^Toxicology Unit, Research Institute of Biomedical and Health Sciences (IUIBS), University of Las Palmas de Gran Canaria (ULPGC), Las Palmas de Gran Canaria, Spain; ^2^Centro de Investigación para la Salud en América Latina (CISeAL), Facultad de Medicina, Pontificia Universidad Católica del Ecuador (PUCE), Quito, Ecuador; ^3^ECUAVOLCAN Research Group, Faculty of Medicine, Pontificia Universidad Católica del Ecuador (PUCE), Quito, Ecuador; ^4^Canary Health Service, Research Unit, University Hospital Nuestra Señora de Candelaria and Primary Care Authority of Tenerife, Santa Cruz de Tenerife, Spain; ^5^Preventive Medicine Department, University Hospital Nuestra Señora de Candelaria, Canary Health Service, Santa Cruz de Tenerife, Spain

**Keywords:** volcanic eruptions, respiratory tract diseases, cardiovascular diseases, eye diseases, skin diseases, mental disorders, public health, environmental exposure

## Abstract

**Background:**

Volcanic eruptions pose substantial health risks to populations living in proximity to active volcanoes, impacting respiratory, cardiovascular, ocular, dermatological, and mental health systems. With a growing number of people globally exposed to volcanic activity, there is a critical need for an interdisciplinary understanding of these health consequences, particularly concerning their pathophysiological mechanisms, epidemiological patterns, and public health implications.

**Methods:**

This scoping review systematically synthesized evidence from studies conducted near 27 volcanoes across diverse geographic regions. The analysis integrated clinical, epidemiological, and public health perspectives to characterize health outcomes and associated risk factors, including geographic location, eruption magnitude, and type of exposure. A novel schematic framework was developed to map pathophysiological mechanisms across multiple organ systems.

**Findings:**

The results indicate that volcanic emissions—such as sulphur dioxide, fine tephra, and volcanic ash—exacerbate pre-existing conditions and contribute to increased morbidity. The most frequently reported health outcomes included acute respiratory symptoms, conjunctivitis, dermatitis, hypertension, and post-traumatic stress disorder. Variability in health impacts was observed across sex, age, and region, with vulnerable groups such as children and older adults disproportionately affected. Chronic exposure was associated with persistent health issues, revealing critical gaps in long-term health surveillance and intervention strategies.

**Conclusion:**

This scoping review highlights the multifaceted health impacts of volcanic eruptions, emphasizing the complex interactions between volcanic emissions and human health. The findings underscore the need for tailored public health interventions, proactive education, and further research to strengthen preparedness and resilience in exposed communities.

## 1 Introduction

Volcanic activity poses substantial public health risks for populations residing in proximity to active or potentially active volcanoes. Globally, there are approximately 1,500 potentially active volcanoes, of which more than 550 have erupted in the past century (1). A significant concentration—over 75%—is distributed along the Pacific Ring of Fire, which spans densely populated regions of Asia and the Americas. Countries such as Indonesia, Japan, the United States, Russia, Chile, and the Philippines account for the highest number of active volcanoes (2).

While volcanoes such as Colima (Mexico), Mount Etna (Italy) (1), and Dukono (Indonesia) are often cited due to frequent activity, other volcanoes—Fuego (Guatemala), Popocatépetl (Mexico), Tungurahua (Ecuador), and Merapi (Indonesia)—have generated eruptions with greater explosivity and more pronounced health and social consequences (3).

The term “volcanic products” refers to a complex mixture of solid, liquid, and gaseous materials expelled during both eruptive and non-eruptive phases. These include primary emissions such as tephra (ash, lapilli, and blocks), pyroclastic flows, lava, and gas plumes composed of sulfur dioxide (SO₂), hydrogen sulfide (H₂S), carbon dioxide (CO₂), hydrochloric acid (HCl), and hydrofluoric acid (HF), among others (4). Chronic emissions during quiescent periods—such as diffuse degassing of CO₂ or radon (^222^Rn)—also pose long-term environmental and health risks (5, 6). The physical and chemical composition of these products, as well as particle size and solubility, critically determine their toxicological profile, and biological uptake. Marine and subaerial volcanoes differ significantly in their emission profiles. Submarine systems, for instance, have been shown to release higher quantities of metal pollutants such as lead, selenium, and mercury due to increased magmatic volatility under hydrostatic pressure (7). These pollutants may bioaccumulate in food chains or contaminate water sources, with repercussions on human health.

Beyond these geophysical drivers, global challenges amplify health risk. Intensifying extreme-weather regimes associated with climate change are expected to modulate secondary volcanic hazards (e.g., rainfall-triggered lahars and ash remobilization), increasing exposure in already vulnerable settlements (8). Social determinants—including rapid, unplanned urban growth, poverty, and low educational attainment—constrain household and system-level preparedness, while gaps in land-use regulation and enforcement allow expansion into high-hazard zones, compounding risk (9).

Between 1,500 and 2017, volcanic activity caused over 278,000 deaths and affected more than 800 million individuals living within 100 km of volcanic centers (10, 11). Epidemiological studies highlight both acute and chronic health outcomes related to exposure. However, few reviews have synthesized this evidence in an integrated, system-based manner. Existing efforts, including foundational work by the International Volcanic Health Hazard Network (IVHHN), have primarily addressed specific health outcomes or isolated events (12).

This scoping review aims to comprehensively assess the health impacts of volcanic activity across five major systems—respiratory, cardiovascular, ocular, dermatological, and mental—focusing on studies involving human populations exposed during both eruptive and non-eruptive periods. Special emphasis is placed on the chemical and physical properties of volcanic products, exposure types, eruption magnitude, and geographic context. By integrating clinical, epidemiological, and environmental health perspectives, this review provides an updated synthesis to inform public health policies, risk mitigation strategies, and future interdisciplinary research.

## 2 Methods

### 2.1 Review design

This review was conducted using the scoping review methodological framework proposed by Arksey and O’Malley (13) and further developed by the Joanna Briggs Institute (JBI) (14). The study protocol was developed in accordance with the PRISMA Extension for Scoping Reviews (PRISMA-ScR) (Supplementary Table 1) (15).

### 2.2 Eligibility criteria

We included peer-reviewed articles assessing the impact of volcanic activity on human health, irrespective of study design (e.g., cross-sectional, cohort, case–control, case reports, ecological studies, and both systematic and non-systematic literature reviews). The inclusion of reviews was justified by the global scope of this study, aimed at comprehensively synthesizing all existing evidence on the health effects of volcanic activity. Studies were selected based on a modified PICO framework (16): Population (P) included individuals or communities residing in volcanic areas; Intervention/Exposure (I) encompassed direct or indirect exposure to volcanic products such as lava, ash, gases, aerosols, and contaminated water; Comparison (C) was not explicitly required given the descriptive nature of the synthesis; Outcomes (O) focused on health effects including respiratory, cardiovascular, dermatological, ocular, and mental health impacts. We excluded studies with unclear exposure descriptions, animal or *in vitro* studies, opinion pieces, and editorials.

### 2.3 Information sources and search strategy

A comprehensive search was conducted in PubMed, MEDLINE, Web of Science, Scopus, Virtual Health Library, Medes, and the British Library database up to August 3, 2023. No language or publication date restrictions were applied. Search terms included combinations of keywords and MeSH terms related to volcanic activity (e.g., “volcanic eruption,” “volcanic gas,” “volcanic ash”) and health outcomes (e.g., “respiratory diseases,” “cardiovascular,” “dermatologic,” “ocular,” “mental health”). The full search strategy is detailed in Supplementary Table 2.

### 2.4 Study selection and data extraction

Articles were screened independently by two reviewers using Rayyan software (www.rayyan.ai). Duplicate records were identified and removed prior to the screening process. Discrepancies were resolved by consensus. A data extraction form was developed and pilot-tested, collecting information on study location, volcano characteristics, eruption type (eruptive or non-eruptive), exposure type (acute or chronic), volcanic products involved (ash, gases, etc.), population characteristics, health outcomes, and study design. Some studies addressed more than one PICO element simultaneously, reflecting multiple exposures and outcomes. These were classified as multicriteria studies by consensus between the reviewers. This classification also included systematic and non-systematic literature reviews, along with a subset of original studies that met multiple PICO criteria, ensuring consistent categorization across heterogeneous sources.

### 2.5 Data analysis

Studies were grouped by health systems affected: respiratory, cardiovascular, ocular, dermatological, and mental health. Data were summarized in tables using Microsoft Excel, documenting eruption year, volcano, country, authors, publication date, objectives, study type, statistical methods, population characteristics, results, pathophysiological causes, and public health implications (Supplementary Table 3). Three team members (J. E. M., D. S. J. A., L. D. M. L.) performed initial filtering and compilation, followed by review and evaluation by two additional members (K. S. R., L. D. B.). Cross-checking and discussions ensured accuracy and relevance (K. S. R., X. S., R. J. S).

Frequencies and percentages were used to describe study characteristics globally; multicriteria studies were counted as single units to avoid overrepresentation in distribution analyses by country and continent. Indeed, to avoid overestimation, the findings reported in systematic and non-systematic reviews (*n* = 11) were not incorporated into the descriptive statistical calculations. For individual disease categories, aggregate proportions were analyzed alongside median, minimum, and maximum values. The mechanistic elements illustrated in the figures were directly extracted from the included source studies, which reported these pathways in association with exposure metrics and clinical outcomes. Visual representations of health effects were developed by the authors using BioRender (www.biorender.com).

### 2.6 Quality appraisal and risk of bias assessment

A risk of bias assessment was conducted as per PRISMA-ScR guidelines (15). However, no articles were excluded due to bias, as this is not mandatory in scoping reviews, the appraisal aimed to systematically map existing research in the field.

## 3 Results

**Figure 1:**
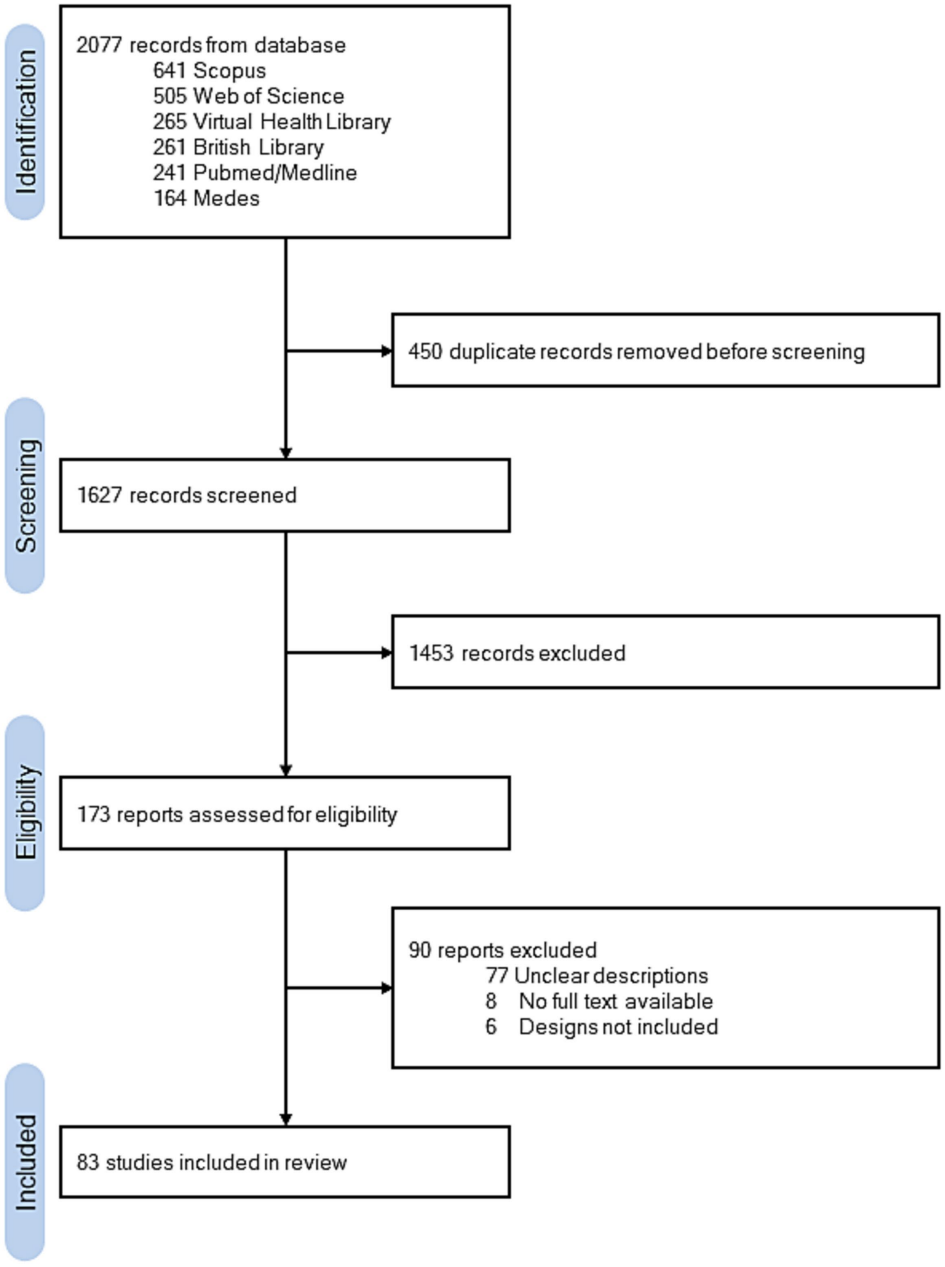
PRISMA flow diagram of the study selection process.

The database search yielded a total of 2,077 records. After the removal of duplicates, 1,627 articles remained for title and abstract screening. This process led to the full-text assessment of 173 studies, with 83 ultimately meeting the inclusion criteria (Figure 1). Articles addressing more than one PICO criterion were classified as multicriteria studies, following consensus among reviewers. The most common reasons for exclusion included inadequate description of exposure, irrelevant outcomes, or unclear study design.

**Figure 2:**
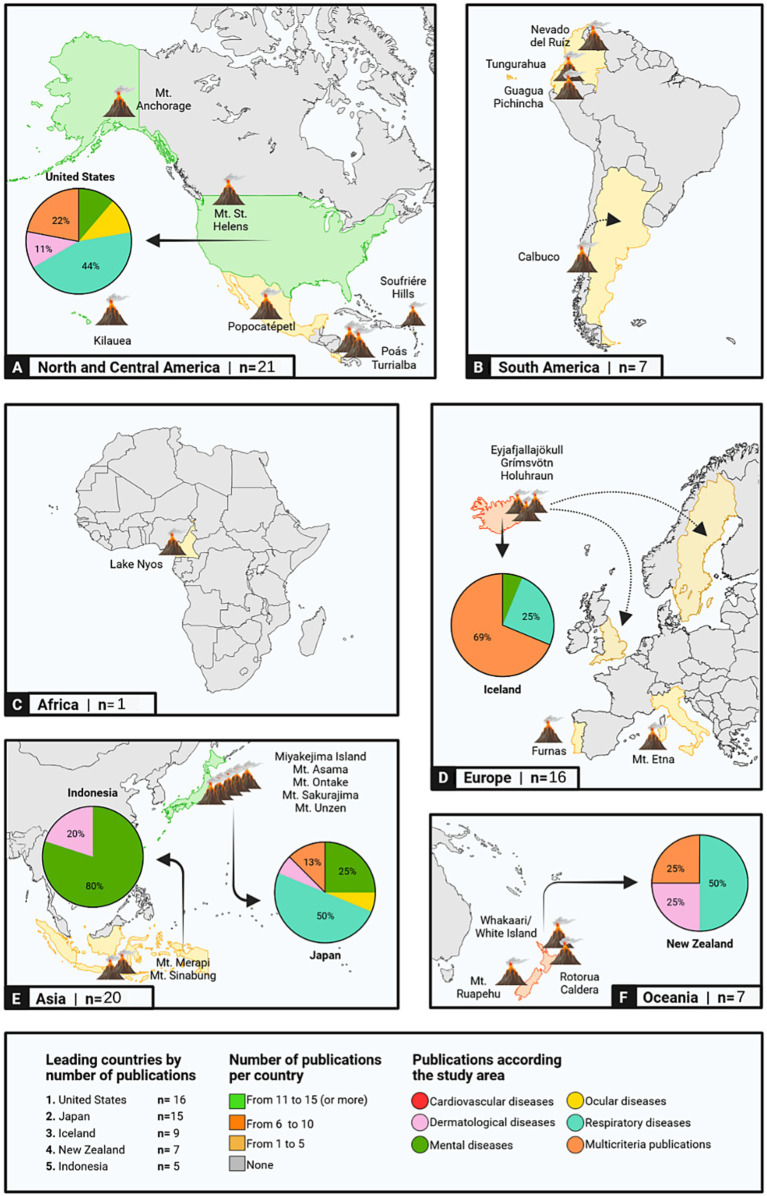
Global geographical distribution of reviewed studies on the health effects of volcanic activity. The figure highlights the number of publications per continent and identifies the top five countries with the highest publication counts within their territories. Pie charts illustrate the percentage distribution of publications across disease categories for each of these top five countries. Additionally, dashed arrows indicate populations affected by eruptions from specific volcanoes and their impact on neighboring nations (This figure was created using Biorender.com).

Geographically, 16 studies (22.22%) were conducted in the United States, followed by Japan with 15 studies (20.83%), and Iceland with 9 studies (12.5%). A total of 27 volcanoes were represented in the literature, with Mount St. Helens (*n* = 8; 11.11%), Kīlauea Volcano (*n* = 7; 9.72%), Eyjafjallajökull Volcano (*n* = 6; 8.33%), and Miyakejima Volcano (*n* = 6; 8.33%) being the most studied (Figure 2). Toxic gases (*n* = 24; 33.33%) and volcanic ash (*n* = 21; 29.17%) were the most frequently assessed volcanic products. The majority of studies focused on adult populations (*n* = 61; 84.72%), with fewer studies investigating children (*n* = 7; 9.72%).

Notable contributors included Carlsen HK, Longo BM, and Baxter PJ, each authoring four studies (4.82%). Peak publication years were 2019 and 2021, with six studies published in each year (7.23%). The earliest included study dated back to 1982.

Five major health domains were identified across the reviewed literature: respiratory, mental health, ocular, dermatological, and cardiovascular effects. Respiratory diseases were the most frequently reported outcomes (*n* = 50; 51.55%), followed by mental health disorders (n = 20; 20.62%) and ocular conditions (*n* = 11; 11.34%). The count of studies by category reflects these multicriteria contributions, thereby ensuring that the synthesis captures the full breadth of volcanic health impacts.

**Figure 3:**
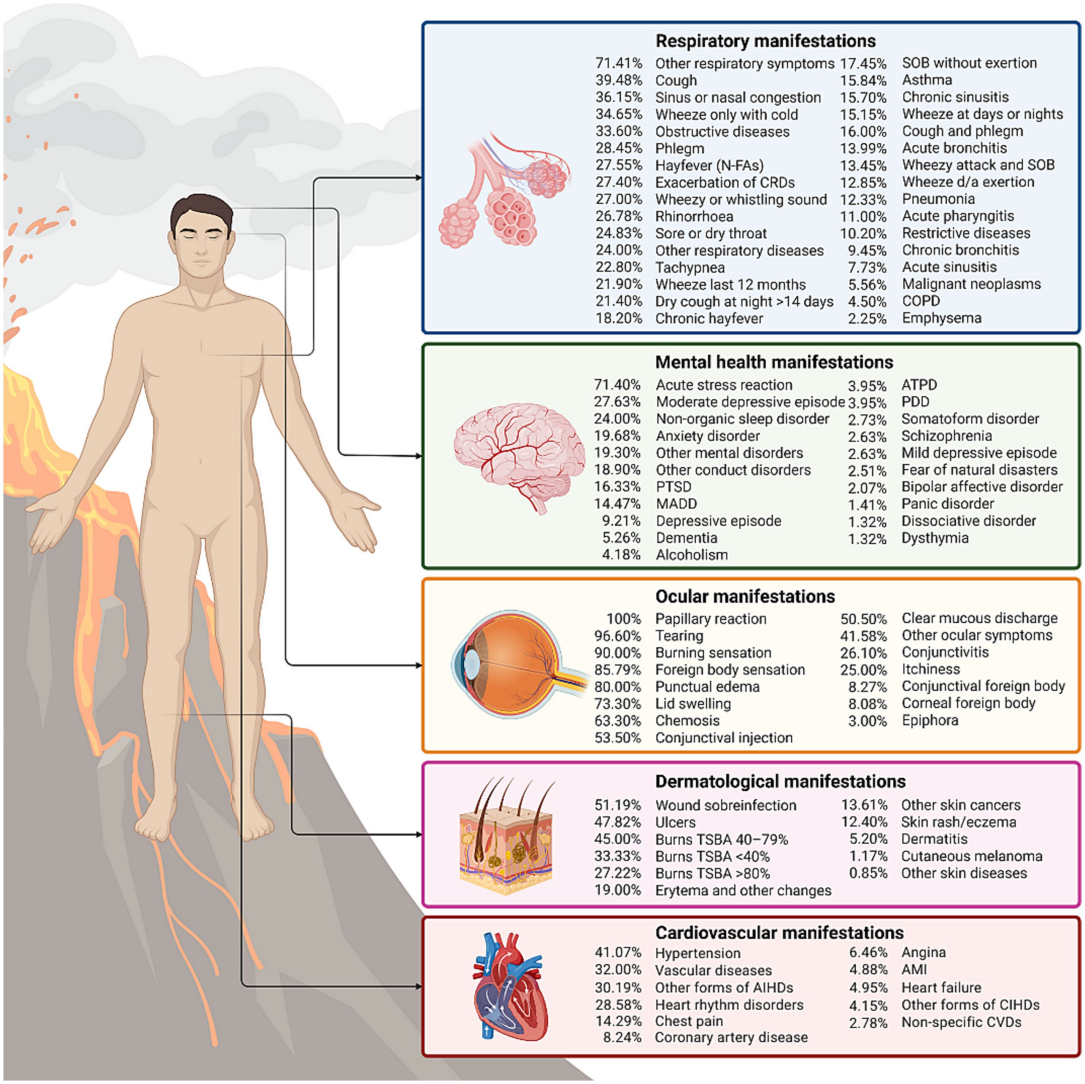
Percentage distribution of multisystemic clinical manifestations associated with volcanic activity exposure, ranked from highest to lowest across each analyzed category. AIHDs, acute ischemic heart diseases; AMI: acute myocardial infarction; ATPD, acute and transient psychotic disorder; CIHDs, chronic ischemic heart diseases; COPD, chronic obstructive pulmonary disease; CRDs, chronic respiratory diseases; d/a, during or after; CVDs: cardiovascular diseases; MADD, mixed anxiety and depression disorder; N-FAs, non-food allergies; PDD, persistent delusional disorder; PTSD, post-traumatic stress disorder; SOB, shortness of breath; TBSA, total body surface area (This figure was created using Biorender.com).

In total, 90 distinct health manifestations were associated with volcanic exposure (Figure 3). Among these, respiratory outcomes comprised 35.56% (*n* = 32), mental health 23.33% (*n* = 21), and ocular effects 16.67% (*n* = 15).

### 3.1 Respiratory effects

This section presents a synthesis of 50 studies that investigated respiratory outcomes associated with volcanic exposure. Of these, 45 were original epidemiological studies (90%) and five were literature reviews (10%) categorized as multicriteria studies. Volcanoes most frequently studied included Kīlauea (*n* = 6; 13.33%), Eyjafjallajökull (*n* = 5; 11.11%), Miyakejima (*n* = 5; 11.11%), Mount St. Helens (*n* = 4; 8.89%), Rotorua Caldera (*n* = 3; 6.67%), Mount Sakurajima (*n* = 3; 6.67%), and 19 other volcanoes of global relevance.

The reported respiratory effects were linked to both the physicochemical properties of volcanic emissions and the exposure context. Ash particles varied in size, morphology, and composition, including crystalline silica, iron, aluminum, copper, and vanadium. Inhalation exposures also involved toxic gases, including carbon monoxide (CO), carbon dioxide (CO₂), sulfur dioxide (SO₂), hydrogen chloride (HCl), hydrogen sulfide (H₂S), and nitrogen dioxide (NO₂), volatile elemental compounds such as diatomic chlorine (Cl₂), elemental sulfur (S₈), and hot water vapor (H₂O), all of which can be harmful to health at high concentrations (17–22). Particles measuring between 2.5 and 10 μm predominantly affected the upper respiratory tract, whereas fine particles (<2.5 μm) penetrated deeply into alveoli (23–25). Sulfurous gases and acidic aerosols showed significant absorption (40–90%) within upper respiratory mucosa, with remaining fractions reaching distal airways via mucociliary clearance.

**Figure 4:**
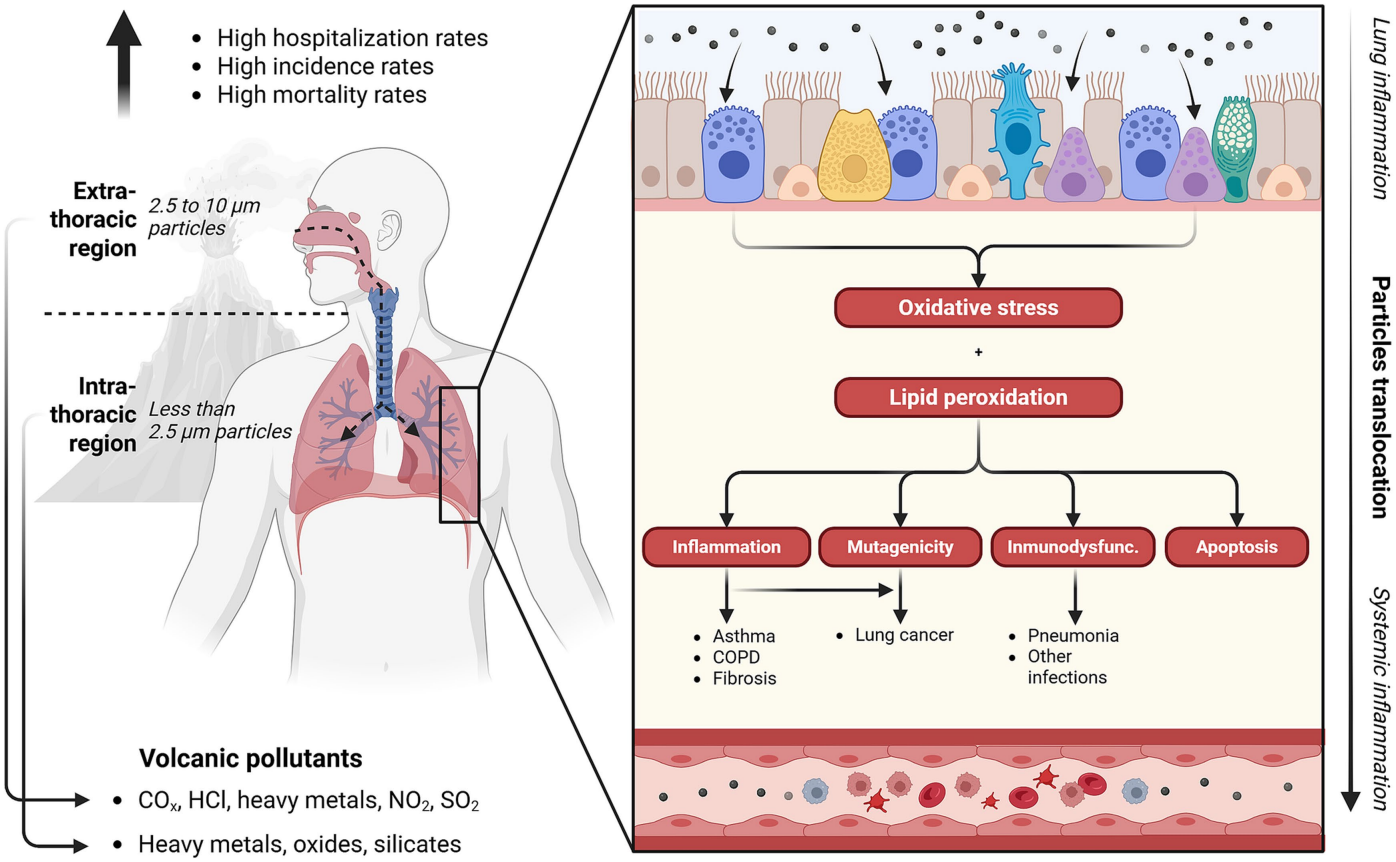
Impact of volcanic activity on respiratory health. COPD, chronic obstructive pulmonary disease; CO_x_, carbon oxides; HCl, hydrochloric acid; immunodysfunc., immunodysfunction; NO_2_, nitrogen dioxide; SO_2_, sulfur dioxide; μm, micrometers. (This figure was created using Biorender.com).

Exposure to these substances at elevated concentrations triggered oxidative stress and lipid peroxidation, initiating complex pathophysiological cascades including epithelial injury, deoxyribonucleic acid (DNA) damage, cell apoptosis, immune dysregulation, and neurogenic inflammation. Chronic inflammatory processes and epithelial barrier disruption increased susceptibility to respiratory infections, alterations in pulmonary microbiota, fibrosis, and impaired pulmonary function. Clinical outcomes reported included asthma, chronic obstructive pulmonary disease (COPD), silicosis, pneumoconiosis, and lung cancer (Figure 4) (17, 18, 20, 21, 24–41). Furthermore, respirable volcanic particles crossing the alveolar-capillary barrier may contribute to systemic pathologies such as cardiovascular and cerebrovascular diseases.

Epidemiological data consistently associated acute exposure to volcanic emissions with increased respiratory morbidities, including cough, sputum production, dyspnea, chest tightness, and wheezing (18, 31, 41–44). Elevated emergency visits due to asthma and COPD exacerbations were documented during eruption events (17, 24–26, 32, 35, 36, 38, 42, 43, 45–48). Severe cases, frequently requiring hospitalization or intensive care, predominantly occurred among older adults and individuals with pre-existing cardiorespiratory conditions, occasionally presenting radiologically as bibasilar atelectasis (18, 29, 36, 49).

Acute respiratory events were particularly pronounced shortly after eruptions (20, 25, 26, 32, 50–53), Acute respiratory events were particularly pronounced shortly after eruptions (26). Conversely, pediatric populations displayed increased susceptibility to upper and lower respiratory tract infections, including sinusitis and bronchitis, particularly among children under 15 years (37, 42, 44, 52, 54). Gender-specific trends emerged, with women exhibiting increased vulnerability to chronic bronchitis and pharyngeal irritation (55).

Comparative analyses between exposed and unexposed cohorts consistently revealed higher incidences of chronic respiratory conditions and symptom exacerbations in ash-impacted areas (25, 30, 32, 40, 44, 47). Symptomatic improvement correlated with decreasing pollutant concentrations over time (27, 47, 48, 56, 57). Some studies suggested increased incidence of respiratory tract cancers (28) and elevated overall mortality rates among exposed populations (20, 41, 46, 58, 59).

Certain studies reported contradictory or inconclusive findings, observing minimal or no significant respiratory outcome differences between exposed and control populations, encompassing both adults and children. Investigations conducted before and after high-level SO_2_ exposure revealed no notable changes in lung function, airway restriction, or inflammatory markers. Such inconsistencies likely reflect methodological limitations, including inadequate sample sizes, short follow-up durations, baseline health disparities, and varying exposure assessments, highlighting the need for more comprehensive, standardized longitudinal studies incorporating detailed biomonitoring (19, 44, 46, 47, 50, 60–66).

### 3.2 Cardiovascular effects

Five epidemiological studies addressing cardiovascular outcomes were identified, conducted in the United States (*n* = 1; 20%), Italy (*n* = 3; 60%), and New Zealand (*n* = 1; 20%). These studies evaluated the health impacts associated with eruptions from Kīlauea Volcano (1983–ongoing), Mount Etna (2002), Mount Ruapehu (1996), and the Colli Albani volcanic complex (quiescent) (17, 21, 46, 67, 68).

Historically, volcanic eruptions were not perceived to significantly impact cardiovascular health acutely (46). In this review, “acute” refers to exposure occurring within eruptive events, while “chronic” denotes persistent exposure to volcanic degassing emissions or prolonged pollutant presence. Recent evidence, however, highlights that acute exposure to elevated concentrations of volcanic ash exacerbates existing cardiovascular conditions, particularly among older adults (17, 67). Specifically, there were significant increases in exacerbations of circulatory system disorders, ischemic heart disease, and acute myocardial infarction by 18, 31, and 34%, respectively. This exposure also correlated with increased frequency and number of cardiovascular-related hospital visits and admissions (17, 67). For instance, Lombardo et al. (17) reported a 1.3% increase in cardiovascular hospital visits following Mount Etna’s eruption between 2001 and 2002 (17). Moreover, elevated mortality risks from ischemic heart disease and myocardial infarction were documented during volcanic eruption periods; however, the reviewed studies did not statistically quantify the magnitude of this risk (67).

Conversely, chronic exposure to volcanic pollutants could trigger a significantly higher prevalence of these diseases (21, 67). Longo et al. (21) assessed the cardiorespiratory impacts of prolonged SO₂ and sulfate aerosols emitted by Kīlauea Volcano, identifying increased prevalence of cardiovascular conditions such as angina, congestive heart failure, and coronary artery disease, particularly among populations exposed to elevated sulfate aerosol concentrations. Statistically significant increased risks were specifically documented for essential hypertension (OR ranging from 1.7 to 2.0) and abnormal blood pressure readings during clinical assessments. Furthermore, an accelerated increase in resting heart rate (mean increase of 8.50 beats/min) was notably prevalent among exposed individuals over 65, non-smokers, and those not receiving cardiovascular medications (21).

Complementing these findings, Carapezza et al. (68) evaluated chronic exposure to endogenous CO₂-rich and H₂S emissions in a residential area of the Metropolitan City of Rome, reporting increased male cardiovascular mortality (HR 1.60, 95% CI 0.95–2.70) and higher risk of myocardial infarction (HR 2.11, 95% CI 0.91–4.90). Comparable risks were also observed in women, underscoring the long-term cardiovascular burden of non-eruptive degassing scenarios (68).

**Figure 5:**
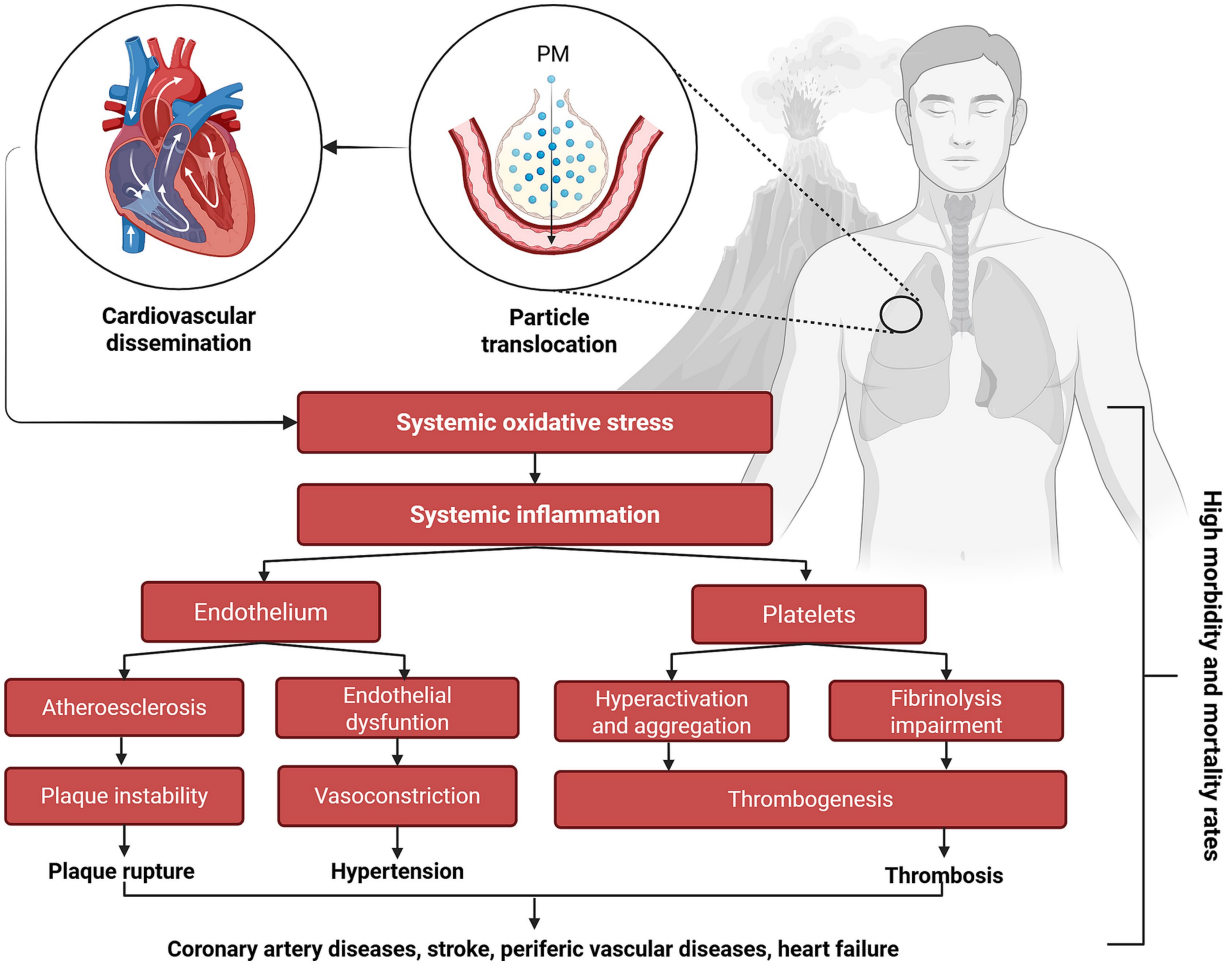
Effects of volcanic activity on cardiovascular health. PM, particulate matter (This figure was created using Biorender.com).

Figure 5 illustrates the mechanistic pathways linking both short- and long-term exposure to volcanic particulate matter (PM_2.5_, PM_10_), toxic volcanic gases (CO_2_, H_2_S, SO_2_), and heavy such as lead (Pb), cadmium (Cd), and arsenic (As) to systemic oxidative stress and chronic inflammation. These pathophysiological processes induce endothelial dysfunction, disrupt autonomic balance, and promote arterial plaque formation, consequently facilitating atherosclerosis development. Additional pathways involve heavy-metal-induced vascular toxicity, resulting in platelet hyperactivation and impaired fibrinolysis. Collectively, these mechanisms amplify risks of developing coronary artery disease, peripheral arterial disease, heart failure, and overall cardiovascular mortality (17, 21, 67).

### 3.3 Ophthalmological effects

This review identified 11 articles addressing ophthalmological impacts associated with volcanic eruptions. The studied volcanoes included Eyjafjallajökull (*n* = 2; 18.18%), Kīlauea (*n* = 2; 18.18%), Miyakejima (*n* = 1; 9.09%), Mount St. Helens (*n* = 1; 9.09%), Poás and Turrialba (*n* = 1; 9.09%), and Sakurajima (*n* = 1; 9.09%), along with three literature reviews (27.27%) that analyzed multiple volcanic events.

**Figure 6:**
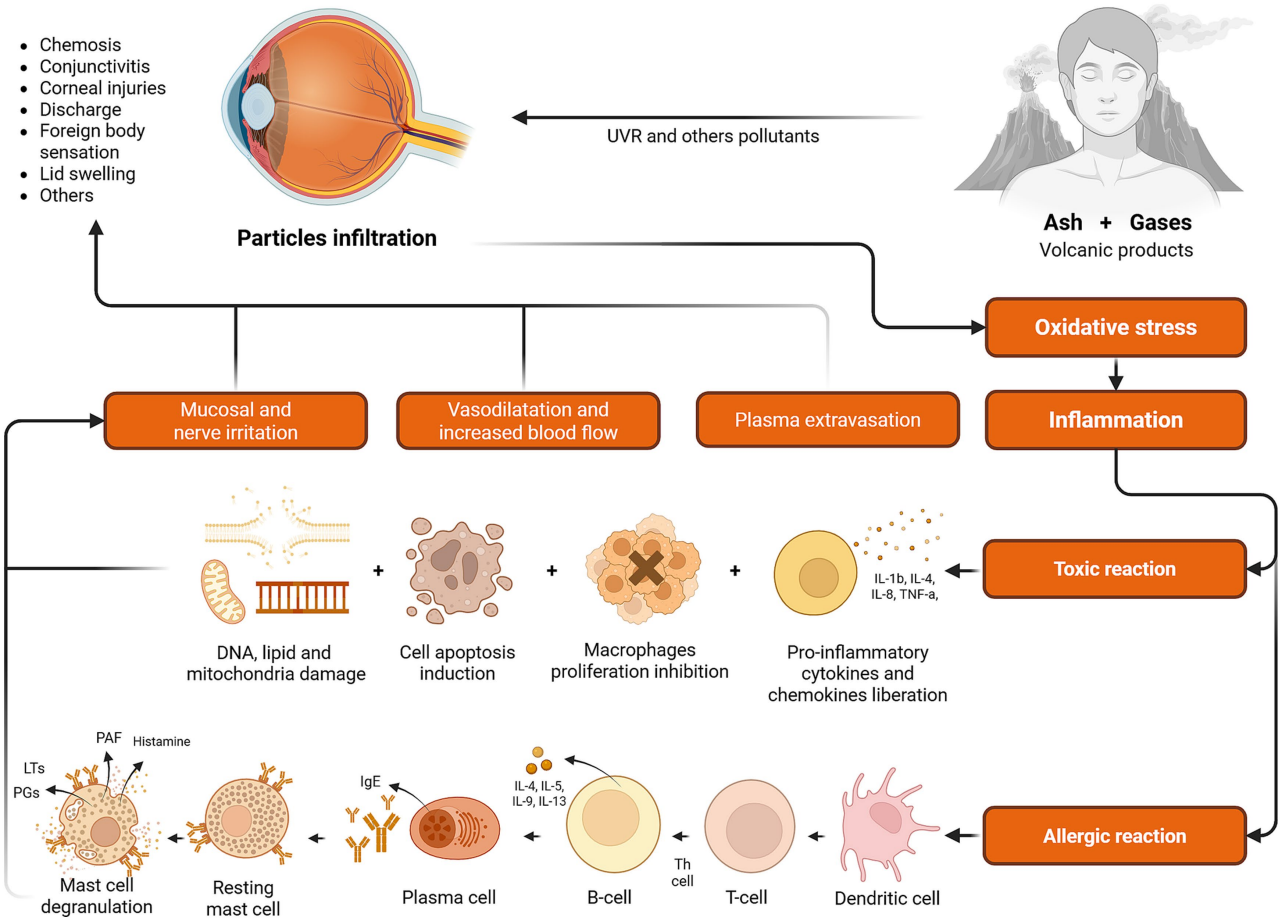
Effects of volcanic activity on ocular health. DNA, deoxyribonucleic acid; IgE, immunoglobulin E; IL, interleukin; LTs, leukotrienes; PAF, platelet-activating factor; PGs, prostaglandins; Th cell, T helper cell; TNF-a, tumor necrosis factor alpha; UVR, ultraviolet radiation (This figure was created using Biorender.com).

Populations exposed to volcanic fog (vog)—a secondary air pollutant formed when SO_2_ and other volcanic gases oxidize and react with atmospheric moisture and sunlight—exhibited numerous ocular manifestations. Commonly reported symptoms included conjunctival injection, clear mucous discharge, papillary reactions, punctate epithelial erosions, eyelid edema, and chemosis, each varying in prevalence based on exposure duration and intensity (69). The ocular conditions termed “vog-induced conjunctivitis” result from both irritative and allergic responses (69). Oxidation of SO₂ produces sulfuric acid (H₂SO₄) aerosols and sulfate particulates, which irritate ocular mucosa and sensory nerves, eliciting tearing, hyperemia, and ocular discomfort. Concurrent allergic mechanisms involve histamine release, exacerbating symptoms such as itching, eyelid swelling, and sub-conjunctival fluid accumulation (Figure 6) (20, 69). For instance, a significant prevalence of ocular irritation (69%) was documented among workers exposed to high atmospheric concentrations of SO₂, H₂S, HCl, HF, and nitric acid (HNO₃) from the Poás and Turrialba volcanoes in Costa Rica (70).

Volcanic ash also poses substantial ocular health risks due to its physical and chemical characteristics, such as particle size distribution, mineralogical composition, and surface reactivity (71, 72). Mechanical abrasion from ash particles accumulating within the conjunctival sac frequently results in corneal epithelial damage and conjunctival inflammation. Even minimal exposure to gaseous volcanic emissions can markedly irritate ocular surfaces (27, 73). These adverse effects can be potentiated by concurrent exposure to ultraviolet radiation, environmental pollutants, and organic solvents. Prolonged corneal epithelial disruption further increases susceptibility to severe corneal infections. Areas with substantial volcanic ash exposure consistently reported higher incidences of ocular complaints, predominantly characterized by conjunctival hyperemia, foreign body sensation, mucopurulent discharge, and pruritus (30, 50, 74). Fraunfelder et al. documented a 72% prevalence of foreign body sensation and conjunctivitis among forestry workers exposed to volcanic ash from Mount St. Helens, highlighting the severe impact of airborne volcanic particulates on ocular health (75).

### 3.4 Dermatological effects

Eleven studies addressing dermatological outcomes associated with volcanic eruptions were analyzed (17, 31, 71, 76–83). Epidemiological research constituted the majority (*n* = 6; 54.55%), supplemented by case series (*n* = 3; 27.27%) and literature reviews classified as multicriteria studies (*n* = 2; 18.18%). Investigations encompassed geographically diverse volcanic eruptions: Mount Etna in Italy (*n* = 2; 18.18%), Whakaari/White Island in New Zealand (*n* = 2; 18.18%), Eyjafjallajökull in Iceland (*n* = 1; 9.09%), Mount Merapi in Indonesia (*n* = 1; 9.09%), Mount Sakurajima in Japan (*n* = 1; 9.09%), Mount St. Helens in the United States (*n* = 1; 9.09%), and Lake Nyos in Cameroon (*n* = 1; 9.09%) (71, 79).

Dermatological effects of volcanic exposure primarily resulted from contact with volcanic ash fallout and gas emissions. Thermal burns represented the predominant acute injury type; however, physical abrasions and chemical burns were also recorded (76, 80). Exposure to hot volcanic gases and ash clouds at extreme temperatures for 60–90 s frequently resulted in severe burns, predominantly affecting exposed skin regions (77, 78, 81). Heat transmission mechanisms included conduction from accumulated ash deposits, convection from heated volcanic clouds, and thermal radiation (78). Burn mortality was substantial, averaging 47.6%, with affected body surface areas ranging from 9 to 90% (76, 78–81). Primary causes of death among burn victims included inhalation injuries and systemic infections predominantly caused by *Aspergillus fumigatus* (76, 78). Notably, elevated sulfate particle concentrations were detected in postmortem skin tissues following the 1980 eruption of Mount St. Helens (81). Recent evidence indicates that volcanic burn injuries necessitated increased surgical intervention time, prolonged hospitalization, and higher healthcare utilization compared to predictive models, reflecting their severity and complexity (76, 80).

**Figure 7:**
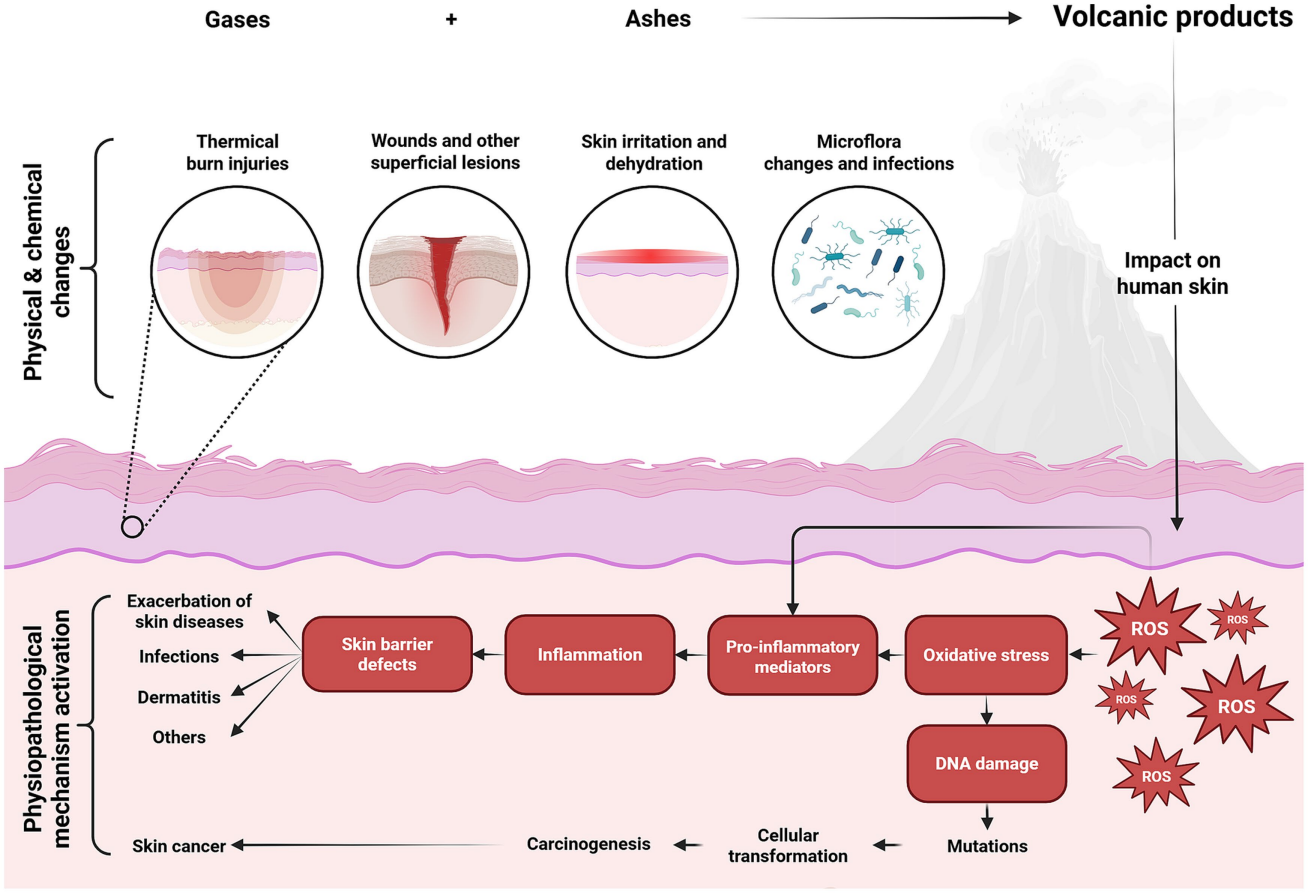
Effects of volcanic activity on skin health. DNA, deoxyribonucleic acid; ROS, reactive oxygen species (This figure was created using Biorender.com).

Chronic dermatological sequelae were significantly more prevalent in volcanically exposed populations compared to non-exposed groups, with an overall increase of 11.60% (31). Frequently observed chronic conditions included eczema, dermatitis, nonspecific eruptions (12.4%), erythema (19%), and ulcerative lesions primarily localized to the face, extremities, and other anatomical regions (47.82%) (76, 80, 82). Bacterial wound infections were common (51.20%), involving pathogens such as *Chryseobacterium indologenes*, *Elizabethkingia miricola*, *Staphylococcus epidermidis*, *Staphylococcus aureus*, and *Streptococcus viridans* (76, 81). Volcanic particulates, characterized by abrasive properties and chemical irritants, promoted oxidative stress, sustained local inflammation, and disrupted skin and mucosal barriers, potentially exacerbating pre-existing dermatological conditions such as eczema, dermatitis, and acne (Figure 7) (31, 71). Additional documented injuries included lacerations, penetrating trauma, compartment syndrome, tetanus, and necrotizing fasciitis (79, 83).

Long-term volcanic ash exposure has also been implicated in dermatological carcinogenesis, with documented cases including melanoma (1.20%) and non-melanoma skin cancers (13.60%) (82). The carcinogenic potential is attributed to chronic inflammatory responses induced by silica-rich ash containing ferric ions and other carcinogenic elements, leading to DNA damage and subsequent malignant transformation (17). Nonetheless, geographical variability and eruption-specific factors influenced the incidence and severity of these dermatological outcomes. For instance, a comparative study following the 2002 Mount Etna eruption revealed no statistically significant difference in dermatological emergency visits between eruption and quiescent periods (17).

### 3.5 Mental health effects

Twenty studies examining the mental health impacts associated with exposure to volcanic ash, gases, and debris were reviewed. The majority comprised epidemiological studies (*n* = 18; 90%), conducted primarily in Iceland (*n* = 5; 27.78%), Indonesia (*n* = 4; 22.22%), Japan (*n* = 4; 22.22%), Colombia (*n* = 3; 16.67%), and the United States (*n* = 2; 11.11%). Volcanic eruptions studied included Eyjafjallajökull (*n* = 4; 22.22%), Mount Merapi (*n* = 3; 16.67%), Nevado del Ruiz (*n* = 3; 16.67%), Mount St. Helens (*n* = 2; 11.11%), Mount Unzen (*n* = 2; 11.11%), among others (*n* = 4; 22.22%). Additionally, two multicriteria literature reviews (10%) addressing mental health impacts were included.

Psychological outcomes were categorized temporally as immediate (e.g., post-traumatic stress disorder (PTSD)), medium-term (e.g., delayed-onset PTSD, major depressive disorder, generalized anxiety disorder), and long-term effects (e.g., psychosomatic disorders) (84–89).

**Figure 8:**
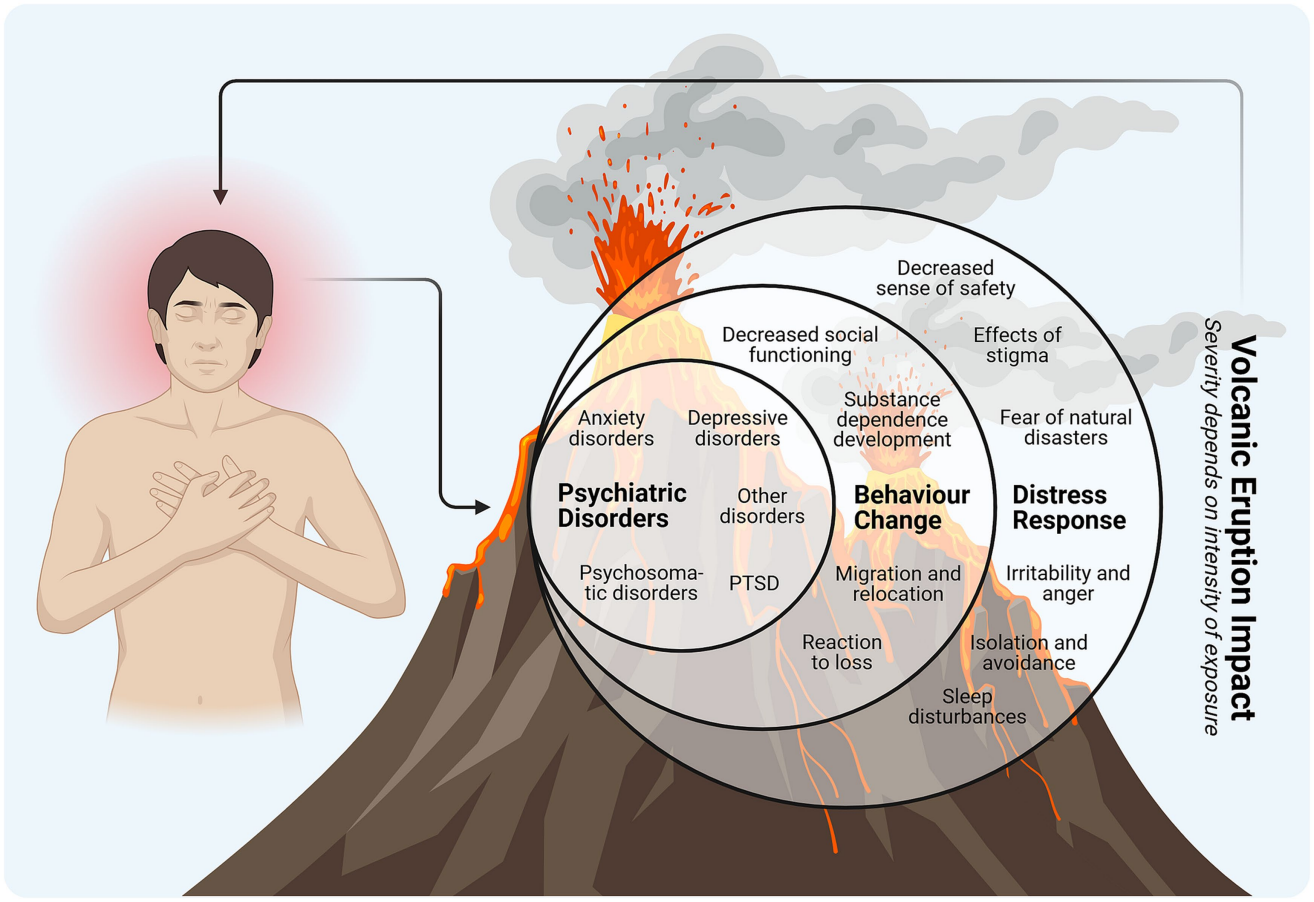
Effects of volcanic activity on mental health. PTSD, post-traumatic stress disorder (This figure was created using Biorender.com).

The most frequently reported mental health diagnoses during intervention periods were prolonged depression (27.63%), PTSD (11.84%), and depressive episodes (9.21%). Less common psychiatric diagnoses included dementia (5.26%), alcohol dependence (3.95%), schizophrenia (2.63%), and various other disorders (84). The incidence of PTSD was notably higher in individuals residing in highly exposed areas or among those reporting multiple physical symptoms related to volcanic exposure (Figure 8) (31, 90–92).

Significant risk factors correlated with higher PTSD incidence included widowhood or divorce, lower socioeconomic status (*p* = 0.033), occupations such as farming or fishing (*p* = 0.011), and pet loss (*p* = 0.002). Additionally, individuals with frequent relocations reported significantly increased PTSD symptoms compared to less frequently relocated individuals. Depressive symptoms strongly correlated with lower socioeconomic status, widowhood, prolonged residence in affected areas, and prior disaster-related evacuation experiences, with older adults particularly affected (87, 91). Persistent physical and psychological symptoms were documented among children, with anxiety levels significantly elevated in moderately exposed (OR = 2.39, 95% CI: 1.67–3.45) and highly exposed (OR = 2.77, 95% CI: 1.81–4.27) groups, underscoring the necessity of targeted preventive and supportive psychological interventions for pediatric populations (42, 86, 93). In the included studies, the terms “low,” “moderate,” and “high exposure,” as well as “frequent relocations,” were operationalized by the original authors, typically according to geographic proximity to the volcano or the number of displacement events, rather than by quantitative exposure metrics.

Gender disparities in mental health outcomes were apparent; generalized anxiety disorder, major depression, and PTSD were more prevalent among women. Compared to low-exposure areas, women in high-exposure areas exhibited an 11-fold increase in risk, whereas men showed a 12.3-fold increase (94).

Additional prevalent psychological symptoms included sadness (83.40%), anger (65.10%), anxiety (42–52%), distress (76.60%), anergia, and social dysfunction (95, 96). However, some studies reported stress, anxiety, or depression symptoms affecting fewer than 10% of individuals in moderately exposed regions (50).

Research addressing mental health impacts following volcanic eruptions remains limited, potentially due to the significant stigma associated with mental health disorders. Such stigma often leads to denial as a coping mechanism among affected populations (30, 85, 97).

Furthermore, mental health outcomes post-eruption are influenced substantially by socioeconomic factors (98), with evidence suggesting that post-disaster relocation accompanied by adequate resources significantly enhances mental health recovery (96, 99).

## 4 Discussion

This review synthesizes evidence regarding the diverse health impacts associated with volcanic eruptions, highlighting significant respiratory, ocular, dermatological, cardiovascular, and mental health outcomes. The severity and prevalence of these effects are influenced by eruption magnitude, exposure type, and local environmental and socio-economic contexts, underscoring the necessity of a holistic approach to evaluating and managing health risks posed by volcanic emissions, including particulate matter such as volcanic ash and toxic gases.

Existing literature reveals that exposure to volcanic emissions, particularly SO_2_, poses significant risks to respiratory, cardiovascular and ocular health (19, 27, 33, 69, 75). Multiple studies have documented a high prevalence of acute conditions such as sinusitis, bronchitis, arterial hypertension, dermatitis, anxiety, PTSD, conjunctivitis, and ocular foreign body sensation among populations exposed to volcanic ash and gases (17, 37, 52, 67, 69–73, 75, 78, 79, 84–87). Chronic exposure to volcanic emissions is particularly concerning, as it fosters sustained inflammatory processes and systemic toxicity. Prolonged contact with SO_2_, sulphate aerosols, heavy metals, and naturally degassing gases, even from quiescent volcanoes, has been linked to progressive respiratory impairment, increased cardiovascular vulnerability, and potential carcinogenic pathways (17, 21, 31, 41, 58, 59, 67, 68, 82). These long-term sequelae often evolve gradually, complicating detection and frequently eluding conventional health surveillance.

Geographical factors critically modulate the observed health impacts among exposed populations. Areas with high volcanic activity report elevated incidences of acute and chronic diseases, highlighting occupational vulnerabilities and the amplifying role of local environmental conditions in health risks (21, 46, 63, 67, 71). Workers regularly exposed to volcanic pollutants show distinct susceptibilities, emphasizing the importance of considering occupational exposure in public health strategies (55, 70). Moreover, eruption frequency and intensity significantly affect symptom prevalence, suggesting heightened risks for populations experiencing repeated or intense volcanic activity (31, 42).

Effective public health management of volcanic hazards requires an integrated approach, combining health monitoring, environmental management, and socio-economic interventions. Respiratory conditions such as asthma and chronic bronchitis, frequently exacerbated by volcanic emissions, place substantial pressure on healthcare systems, a phenomenon notably evident during crises such as the COVID-19 pandemic, and likely applicable to communities near active volcanoes (31). Vulnerable groups, particularly children, older adults, and individuals with pre-existing medical conditions, exhibit heightened susceptibility, underscoring the critical need for targeted monitoring and robust public health interventions to mitigate long-term morbidity (29, 30).

Beyond respiratory concerns, volcanic emissions significantly compromise water and air quality, impacting food security and community health. Contamination with heavy metals (e.g., lead, mercury, arsenic) linked to volcanic activity poses substantial health risks, including neurodevelopmental impairments, cancer, thyroid disruption, immunosuppression, and malnutrition, especially in developing countries with limited healthcare infrastructure (100–102). These environmental health threats necessitate rigorous monitoring and targeted mitigation strategies, including improvements in water and food quality, enhanced sanitation systems, timely communication of volcanic activity and comprehensive volcano monitoring to safeguard community health, particularly among vulnerable populations such as children (103).

Forced displacement due to volcanic hazards further exacerbates health outcomes, affecting mental health, substance use, and perinatal health (104–106). Evidence increasingly suggests that traumatic events, including forced relocations, directly influence health outcomes. Displacement disrupts livelihoods, social support networks, and cultural continuity, amplifying existing health disparities and straining healthcare resources (107, 108). Comprehensive public health interventions addressing social, mental, and physical health, combined with sustainable economic support and community-driven resilience-building programs, are essential for mitigating these impacts.

Public education, preparedness, and policy interventions play crucial roles in reducing health risks associated with volcanic emissions. Early warning systems, protective equipment guidelines, regular emergency drills, effective communication strategies, and accessible emergency shelters are integral components for enhancing community resilience and rapid response capabilities (109, 110). Policies emphasizing continuous air quality monitoring, targeted support for vulnerable groups, and sustainable funding for preventive infrastructure substantially strengthen community preparedness and healthcare system resilience against volcanic hazards (111).

### 4.1 Limitations of the study

This review acknowledges certain limitations, notably the heterogeneity of study designs, complicating direct comparisons. The absence of a systematic risk-of-bias assessment could affect the robustness of conclusions, although such assessments are not mandatory for scoping reviews. Although meta-analysis was not feasible due to methodological diversity, the synthesis allows for the identification of trends and priority areas for future research. In this study, some pollutants of high toxicological relevance, such as fluoride, were not considered, as their primary effects involve the skeletal system, which was beyond the scope of this review. Other organ systems potentially affected by volcanic emissions, also did not emerge in the selected literature. Furthermore, exposure levels were often defined indirectly rather than measured concentrations, limiting dose–response inference. Nevertheless, findings presented offer valuable insights into volcanic eruption health impacts, highlighting the necessity for standardized methodologies and sustained health monitoring to improve comparability and reliability across studies.

### 4.2 Recommendation for further research

Further research should elucidate how volcanic products enter the body and their associated physiological, biochemical, and immunological impacts, particularly regarding cardiovascular health. Targeted studies are needed for vulnerable groups, including children, pregnant women, older adults, and displaced populations in high-risk areas. Moreover, research should expand beyond currently studied systems to include metabolic, hepatic, nephrological, reproductive, and skeletal outcomes, and evaluate emergent burdens such as cancer, neurodegenerative and maternal–child health disorders. Likewise, incorporating grey literature and operational reports would complement peer-reviewed evidence, thereby enhancing contextual understanding and guiding preparedness strategies. The integration of social determinants of health is essential to ensure the development of interventions tailored to community needs.

## 5 Conclusion

This scoping review provides a comprehensive overview of the health impacts associated with volcanic eruptions, highlighting the complex interactions between volcanic emissions and human health, particularly in communities near active volcanoes. Health consequences frequently result from direct exposure to volcanic ash and gases or indirect contact through polluted air and water sources. Notably, volcanic emissions can aggravate pre-existing medical conditions and elevate morbidity rates, with variability in health outcomes observed according to demographic factors such as age and sex. These findings underscore the necessity for tailored, evidence-based interventions, proactive public health education, and further research to enhance community preparedness, resilience, and reduce long-term health vulnerabilities.

## Data Availability

The published article and supplemental information include all data generated and analysed during this study. Any additional information required to reanalyse the data reported in this paper is available from the lead contact, Katherine Simbaña-Rivera (katherine.simbana101@alu.ulpgc.es), upon request.
