# Supplementary material for: Mapping the global health burden of volcanic exposure: a scoping review approach
Source: Front Public Health. 2025 Sep 24;13:1658384. doi: 10.3389/fpubh.2025.1658384 (PMC12504282; doi:10.3389/fpubh.2025.1658384)
Supplement: Supplementary file 1 [file Data_Sheet_1.docx]

***Supplementary Material***

# Supplementary tables

## Supplementary Table 1: Search strategy.

The search was conducted between July 19, 2023, and August 3, 2023. The search string adapted to each database functionality was the following for each affected system:

| **Database** | **Search Terms** | **Results** |
| --- | --- | --- |
| Pubmed/Medline | ((Volcanic Ash OR Volcanic Eruption OR Volcanic Gases)[MeSH Terms]) AND (Respiratory Tract Diseases OR Disease, Respiratory Tract OR Respiratory System Disease OR Respiratory Diseases)[MeSH Terms]) | 123 |
| Pubmed/Medline | (((Volcanic Ash OR Volcanic Eruption OR Volcanic Gases)[MeSH Terms])) AND ((Cardiac Toxicity OR cardio toxicities OR Circulatory System OR Cardiovascular Diseases OR Vascular Endothelium OR Capillary Endothelium)[MeSH Terms])) | 30 |
| Pubmed/Medline | (Volcanic Eruptions[MeSH Terms] OR Volcanic Ash OR Volcanic Eruption OR Volcanic Gases) AND ((Dry Eye Syndrome[MeSH Terms]) OR (Dry Eye Disease[MeSH Terms]) OR (Dry Eye Diseases[MeSH Terms]) OR (Dry Eyes[MeSH Terms]) OR (Keratoconjunctivitis[MeSH Terms]) OR (Conjunctivitis, Allergic[MeSH Terms]) OR (Keratoconjunctivitis Sicca[MeSH Terms]) OR (Conjunctival Disease[MeSH Terms]) OR (Eye Diseases[MeSH Terms]) OR (Eye Injury[MeSH Terms]) OR (Eye Abnormalities[MeSH Terms]) OR (Corneal Injuries[MeSH Terms]) OR (Eye Neoplasms[MeSH Terms]) OR (CANCER OF EYE) OR (EYE CANCER)) | 8 |
| Pubmed/Medline | (Skin Disease OR Dermatosis OR Skin and Subcutaneous Tissue Disorders OR Dermatitis OR Burns) AND ((Volcanic Ash OR Volcanic Eruption OR Volcanic Gases)) | 63 |
| Pubmed/Medline | (Volcanic Eruptions[MeSH Terms]) AND ((Mental Health[MeSH Terms]) OR (Anxiety[MeSH Terms]) OR (Depression[MeSH Terms]) OR (Stress Disorders, Post-Traumatic[MeSH Terms]) OR (Stress Disorders, Traumatic, Acute[MeSH Terms]) OR (Stress, Psychological[MeSH Terms]) OR (Suicidal Ideation[MeSH Terms]) OR (Sleep Initiation and Maintenance Disorders[MeSH Terms])) | 17 |
| Scopus | (ALL (respiratory tract diseases) AND ALL (inhalation) AND ALL (volcanic ash) AND ALL (risk)) | 154 |
| Scopus | (ALL (cardiac toxicity) OR ALL (cardiovascular diseases) OR ALL (vascular endothelium) AND ALL (inhalation) AND ALL (volcanic ash) AND ALL (risk)) | 145 |
| Scopus | (ALL (eye diseases) OR TITLE-ABS-KEY (dry eye syndrome) OR TITLE-ABS-KEY (keratoconjunctivitis) OR TITLE-ABS-KEY (dry eye disease) OR TITLE-ABS-KEY (conjunctivitis allergic) OR TITLE-ABS-KEY (keratoconjunctivitis AND sicca) OR TITLE-ABS-KEY (conjunctival disease) OR TITLE-ABS-KEY (eye injury) OR TITLE-ABS-KEY (eye abnormalities) OR TITLE-ABS-KEY (corneal injuries) OR TITLE-ABS-KEY (eye neoplasms) OR TITLE-ABS-KEY (cancer of eye) AND ALL (volcanic ash OR volcanic eruption OR volcanic gases)) | 264 |
| Scopus | (ALL (volcanic ash) OR TITLE-ABS-KEY (volcanic eruption) AND TITLE-ABS-KEY (skin disease)) | 40 |
| Scopus | INDEXTERMS (volcanic eruptions) AND (INDEXTERMS (mental health) OR INDEXTERMS (anxiety) OR INDEXTERMS (depression) OR INDEXTERMS (stress disorders AND post-traumatic) OR INDEXTERMS (suicidal ideation) OR INDEXTERMS (stress psychological) OR INDEXTERMS (stress disorders traumatic acute) OR INDEXTERMS (sleep initiation maintenance disorders)) | 38 |
| Web of Science | (ALL=((Volcanic Ash OR Volcanic Eruption OR Volcanic Gases))) AND ALL=((Respiratory Tract Diseases OR Disease, Respiratory Tract OR Respiratory System Disease OR Respiratory Diseases)) | 70 |
| Web of Science | (ALL=((Volcanic Ash OR Volcanic Eruption OR Volcanic Gases))) AND ALL=((Cardiac Toxicity OR Cardiotoxicities OR Circulatory System OR Cardiovascular Diseases OR Vascular Endothelium OR Capillary Endothelium)) | 17 |
| Web of Science | (ALL=((Volcanic Ash OR Volcanic Eruption OR Volcanic Gases))) AND ALL=(Dry Eye Syndrome OR Dry Eye Disease OR Dry Eye Diseases OR Dry Eyes OR Keratoconjunctivitis OR Conjunctivitis, Allergic OR Keratoconjunctivitis Sicca OR Conjunctival Disease OR Eye Diseases OR Eye Injury OR Eye Abnormalities OR Corneal Injuries OR Eye Neoplasms OR CANCER OF EYE OR EYE CANCER) | 8 |
| Web of Science | (ALL=((Volcanic Ash OR Volcanic Eruption OR Volcanic Gases))) AND ALL=(Skin Disease OR Dermatosis OR Skin and Subcutaneous Tissue Disorders OR Dermatitis OR Burns) | 352 |
| Web of Science | (ALL=(Volcanic Eruptions)) AND (ALL=("Mental Health") OR KP=(Anxiety) OR KP=(Depression) OR KP=(Stress Disorders, Post-Traumatic) OR KP=(Suicidal Ideation) OR KP=(Stress, Psychological) OR KP=(Stress Disorders, Traumatic, Acute) OR KP=(Sleep Initiation and Maintenance Disorders)) | 58 |
| Virtual Health  Library | ((Volcanic Eruptions) OR (Volcanic Gases) OR (Volcanic Ash) OR (Erupções Vulcânicas) OR (Erupciones Volcánicas)) AND ((Respiratory Tract Diseases) OR (Doenças do trato respiratório) OR (Enfermedades del tracto respiratorio ) OR (Disease, Respiratory Tract) OR (Respiratory System Disease) OR (Doenças do sistema respiratório) OR (Enfermedades del sistema respiratorio) OR (Respiratory Diseases) OR (Doenças respiratórias) OR (Enfermedades respiratorias)) | 100 |
| Virtual Health  Library | ((Volcanic Eruptions) OR (Volcanic Gases) OR (Volcanic Ash) OR (Erupções Vulcânicas) OR (Erupciones Volcánicas)) AND ((Cardiac Toxicity) OR (Toxicidade cardíaca) OR (Toxicidad cardiaca) OR (Cardiotoxicities) OR (Cardiotoxicidades) OR (Circulatory System) OR (Sistema circulatorio) OR (Sistema circulatório) OR (Cardiovascular Diseases) OR (Enfermedades cardiovasculares) OR (Doenças cardiovasculares) OR (Vascular Endothelium) OR (Endotelio vascular) OR (Endotélio vascular) OR (Capillary Endothelium) OR (Endotelio capilar) OR (Endotélio capilar)) | 21 |
| Virtual Health  Library | ((Volcanic Eruptions) OR (Volcanic Gases) OR (Volcanic Ash) OR (Erupções Vulcânicas) OR (Erupciones Volcánicas)) AND ((Dry Eye Syndrome) OR (Dry Eye Disease) OR (Dry Eye Diseases) OR (Dry Eyes) OR (Keratoconjunctivitis) OR (Conjunctivitis, Allergic) OR (Keratoconjunctivitis Sicca) OR (Conjunctival Disease) OR (Eye Diseases) OR (Eye Injury) OR (Eye Abnormalities) OR (Corneal Injuries) OR (Eye Neoplasms) OR (CANCER OF EYE) OR (EYE CANCER) OR (Conjuntivitis) OR (Blefaritis) OR (Orzuelo) OR (QUEMADURA CORNEAL) OR (ULCERAS CORNEALES) OR (Síndromes de Ojo Seco) OR (Lesiones de la Cornea) OR (Abrasiones Corneales) OR (Queratoconjuntivitis) OR (Conjuntivitis Alérgica) OR (QUERATOCONJUNTIVITIS QUIMICA) OR (Anomalías del Ojo) OR (Neoplasias del Ojo) OR (Conjuntivite) OR (Blefarite) OR (Terçol) OR (QUEIMADURA DA CÓRNEA) OR (ÚLCERAS DE CÓRNEA) OR (Síndromes do Olho Seco) OR (Lesões da Córnea) OR (Ceratoconjuntivite) OR (CERATOCONJUNTIVITE QUÍMICA) OR (Conjuntivite Alérgica) OR (Anormalidades do Olho) OR (Neoplasias Oculares)) | 17 |
| Virtual Health  Library | (((Volcanic Eruptions) OR (Volcanic Gases) OR (Volcanic Ash) OR (Erupções Vulcânicas) OR (Erupciones Volcánicas)) AND ((Skin Disease) OR (Enfermedad de la piel) OR (Doenças da pele) OR (Dermatosis) OR (Dermatose) OR (Skin and Subcutaneous Tissue Disorders) OR (Trastornos de la piel y del tejido subcutáneo) OR (Perturbações da pele e do tecido subcutâneo) OR (Dermatitis) OR (Dermatite) OR (Burns) OR (Quemaduras) OR (Queimaduras))) | 29 |
| Virtual Health  Library | ((Volcanic Eruptions) OR (Volcanic Gases) OR (Volcanic Ash) OR (Erupções Vulcânicas) OR (Erupciones Volcánicas)) AND ((Mental Health) OR (Salud Mental) OR (Saúde Mental) OR (Anxiety) OR (Ansiedad) OR (Ansiedade) OR (Depression) OR (Depressão) OR (Depresión) OR (Stress Disorders, Post-Traumatic) OR (Trastornos por Estrés Postraumático) OR (Transtornos de Estresse Pós-Traumáticos) OR (Stress Disorders, Traumatic, Acute) OR (Trastornos de Estrés Traumático Agudo) OR (Transtornos de Estresse Traumático Agudo) OR (Stress, Psychological) OR (Estrés Psicológico) OR (Estresse Psicológico) OR (Suicidal Ideation) OR (Ideación Suicida) OR (Ideação Suicida) OR (Sleep Initiation and Maintenance Disorders) OR (Trastornos del Inicio y del Mantenimiento del Sueño) OR (Distúrbios do Início e da Manutenção do Sono)) | 98 |
| Medes | ((“Volcanic Eruptions”[título/resumen/palabras_clave]) OR (“Volcanic Gases”[título/resumen/palabras_clave]) OR (“Volcanic Ash”[título/resumen/palabras_clave]) OR (“Erupções Vulcânicas”[título/resumen/palabras_clave]) OR (“Erupciones Volcánicas”[título/resumen/palabras_clave])) AND (("Respiratory Tract Diseases"[todos]) OR ("Doenças do trato respiratório"[todos]) OR ("Enfermedades del tracto respiratorio"[todos]) OR ("Disease, Respiratory Tract"[todos]) OR ("Respiratory System Disease"[todos]) OR ("Doenças do sistema respiratório"[todos]) OR ("Enfermedades del sistema respiratorio"[todos]) OR ("Respiratory Diseases"[todos]) OR ("Doenças respiratórias"[todos]) OR ("Enfermedades respiratorias"[todos])) | 0 |
| Medes | ((((((((("Volcanic Eruptions"[todos]) OR "Volcanic Gases"[todos]) OR "Volcanic Ash"[todos]) OR "Erupções Vulcânicas"[todos]) OR "Erupciones Volcánicas"[todos])) AND "Cardiac Toxicity"[todos]) OR "Toxicidade cardíaca"[todos]) OR "Cardiotoxicidades"[todos]) | 37 |
| Medes | ((“Dry Eye Syndrome”[título/resumen/palabras_clave]) OR (“Dry Eye Disease”[título/resumen/palabras_clave]) OR (“Dry Eye Diseases”[título/resumen/palabras_clave]) OR (“Dry Eyes”[título/resumen/palabras_clave]) OR (“Keratoconjunctivitis”[título/resumen/palabras_clave]) OR (“Conjunctivitis, Allergic”[título/resumen/palabras_clave]) OR (“Keratoconjunctivitis Sicca”[título/resumen/palabras_clave]) OR (“Conjunctival Disease”[título/resumen/palabras_clave]) OR (“Eye Diseases”[título/resumen/palabras_clave]) OR (“Eye Injury”[título/resumen/palabras_clave]) OR (“Eye Abnormalities”[título/resumen/palabras_clave]) OR (“Corneal Injuries”[título/resumen/palabras_clave]) OR (“Eye Neoplasms”[título/resumen/palabras_clave]) OR (“CANCER OF EYE”[título/resumen/palabras_clave]) OR (“EYE CANCER”[título/resumen/palabras_clave]) OR (“Conjuntivitis”[título/resumen/palabras_clave]) OR (“Blefaritis”[título/resumen/palabras_clave]) OR (“Orzuelo”[título/resumen/palabras_clave]) OR (“QUEMADURA CORNEAL”[título/resumen/palabras_clave]) OR (“ULCERAS CORNEALES”[título/resumen/palabras_clave]) OR (“Síndromes de Ojo Seco”[título/resumen/palabras_clave]) OR (“Lesiones de la Cornea”[título/resumen/palabras_clave]) OR (“Abrasiones Corneales” [título/resumen/palabras_clave]) OR (“Queratoconjuntivitis” [título/resumen/palabras_clave]) OR (“Conjuntivitis Alérgica”[título/resumen/palabras_clave]) OR (“QUERATOCONJUNTIVITIS QUIMICA”[título/resumen/palabras_clave]) OR (“Anomalías del Ojo”[título/resumen/palabras_clave]) OR (“Neoplasias del Ojo”[título/resumen/palabras_clave]) OR (“Conjuntivite”[título/resumen/palabras_clave]) OR (“Blefarite”[título/resumen/palabras_clave]) OR (“Terçol”[título/resumen/palabras_clave]) OR (“QUEIMADURA DA CÓRNEA”[título/resumen/palabras_clave]) OR (“ÚLCERAS DE CÓRNEA” [título/resumen/palabras_clave]) OR (“Síndromes do Olho Seco”[título/resumen/palabras_clave]) OR (“Lesões da Córnea”[título/resumen/palabras_clave]) OR (“Ceratoconjuntivite”[título/resumen/palabras_clave]) OR (“Conjuntivite Alérgica”[título/resumen/palabras_clave]) OR (“CERATOCONJUNTIVITE QUÍMICA”[título/resumen/palabras_clave]) OR (“Anormalidades do Olho”[título/resumen/palabras_clave]) OR (“Neoplasias Oculares”[título/resumen/palabras_clave])) AND ((“Volcanic Eruptions”[título/resumen/palabras_clave]) OR (“Volcanic Gases”[título/resumen/palabras_clave]) OR (“Volcanic Ash”[título/resumen/palabras_clave]) OR (“Erupções Vulcânicas”[título/resumen/palabras_clave]) OR (“Erupciones Volcánicas”[título/resumen/palabras_clave])) | 75 |
| Medes | ((((((((("Volcanic Eruptions"[todos]) OR "Volcanic Gases"[todos]) OR "Volcanic Ash"[todos]) OR "Erupções Vulcânicas"[todos]) OR "Erupciones Volcánicas"[todos])) AND "Skin Disease"[todos]) OR "Enfermedad de la piel"[todos]) OR "Doenças da pele"[todos]) | 6 |
| Medes | ((“Volcanic Eruptions”[título/resumen/palabras_clave]) OR (“Volcanic Gases”[título/resumen/palabras_clave]) OR (“Volcanic Ash”[título/resumen/palabras_clave]) OR (“Erupções Vulcânicas”[título/resumen/palabras_clave]) OR (“Erupciones Volcánicas”[título/resumen/palabras_clave])) AND ((“Mental Health”[título/resumen/palabras_clave]) OR (“Salud Mental”[título/resumen/palabras_clave]) OR (“Saúde Mental”[título/resumen/palabras_clave]) OR (“Anxiety”[título/resumen/palabras_clave]) OR (“Ansiedad”[título/resumen/palabras_clave]) OR (“Ansiedade”[título/resumen/palabras_clave]) OR (“Depression”[título/resumen/palabras_clave]) OR (“Depressão”[título/resumen/palabras_clave]) OR (“Depresión”[título/resumen/palabras_clave]) OR (“Stress Disorders, Post-Traumatic”[título/resumen/palabras_clave]) OR (“Trastornos por Estrés Postraumático”[título/resumen/palabras_clave]) OR (“Transtornos de Estresse Pós-Traumáticos”[título/resumen/palabras_clave]) OR (“Stress Disorders, Traumatic, Acute”[título/resumen/palabras_clave]) OR (“Trastornos de Estrés Traumático Agudo”[título/resumen/palabras_clave]) OR (“Transtornos de Estresse Traumático Agudo”[título/resumen/palabras_clave]) OR (“Stress, Psychological”[título/resumen/palabras_clave]) OR (“Estrés Psicológico”[título/resumen/palabras_clave]) OR (“Estresse Psicológico”[título/resumen/palabras_clave]) OR (“Suicidal Ideation”[título/resumen/palabras_clave]) OR (“Ideación Suicida”[título/resumen/palabras_clave]) OR (“Ideação Suicida”[título/resumen/palabras_clave]) OR (“Sleep Initiation and Maintenance Disorders”[título/resumen/palabras_clave]) OR (“Trastornos del Inicio y del Mantenimiento del Sueño”[título/resumen/palabras_clave]) OR (“Distúrbios do Início e da Manutenção do Sono”[título/resumen/palabras_clave])) | 46 |
| British Library | (Respiratory Tract Diseases OR Disease, Respiratory Tract OR Respiratory System Disease OR Respiratory Diseases) AND (Volcanic Ash OR Volcanic Eruption OR Volcanic Gases) | 31 |
| British Library | (Cardiac Toxicity OR Cardiotoxicities OR Circulatory System OR Cardiovascular Diseases OR Vascular Endothelium OR Capillary Endothelium) AND (Volcanic Ash OR Volcanic Eruption OR Volcanic Gases) | 10 |
| British Library | (Volcanic Eruptions OR Volcanic Ash OR Volcanic Eruption OR Volcanic Gases) AND (Dry Eye Syndrome OR Dry Eye Disease OR Dry Eye Diseases OR Dry Eyes OR Keratoconjunctivitis OR Conjunctivitis, Allergic OR Keratoconjunctivitis Sicca OR Conjunctival Disease OR Eye Diseases OR Eye Injury OR Eye Abnormalities OR Corneal Injuries OR Eye Neoplasms OR CANCER OF EYE OR EYE CANCER) | 14 |
| British Library | (Volcanic Ash OR Volcanic Eruption OR Volcanic Gases) AND (Skin Disease OR Dermatosis OR Skin and Subcutaneous Tissue Disorders OR Dermatitis OR Burns) | 25 |
| British Library | ((Volcanic Eruptions) OR (Volcanic Gases) OR (Volcanic Ash)) AND ((Mental Health) OR (Anxiety) OR (Depression) OR (Stress Disorders, Post-Traumatic) OR (Stress Disorders, Traumatic, Acute) OR (Stress, Psychological) OR (Suicidal Ideation) OR (Sleep Initiation and Maintenance Disorders)) | 181 |

### Supplementary Table 2:

### Supplementary Table 2a: Data extraction and synthesis of findings on respiratory diseases (n=50).

| **Author, Year** | **Country** | **Volcano** | **State of activity** | **Volcanic product (dimension)** | **Year of eruption** | **Population** | **Study design** | **Findings** |
| --- | --- | --- | --- | --- | --- | --- | --- | --- |
| Lombardo D, et al. 2013† | Italy | Etna | Erupting volcano | Ash (not reported) | 2002 | General population | Epidemiological study | Significant increase of ED visits for acute respiratory and cardiovascular diseases, and ocular disturbances during the ash exposure time. |
| Hickling J, et al. 1999† | New Zealand | Ruapehu | Erupting volcano | Ash (>0.25 mm) | 1996 | General population | Epidemiological study | The study found a protective effect of volcanic eruptions on two types of IHD, “Other acute IHD” and angina pectoris. Exposure to volcanic ashfall from Mt. Ruapehu eruption led to a 44% increase in hospital admissions for acute bronchitis. Previous studies, including Mt. St. Helens reported transient increases in respiratory symptoms like irritation, congestion, asthma, and acute bronchitis, but no new cases of ash-related asthma. Additionally, volcanic eruptions caused significant anxiety among affected populations. |
| Nieto-Torres A, et al. 2021 | Mexico | Popocatepetl | Erupting volcano | Ash (not reported) | 1994-2008 | General population | Epidemiological study | The study found that ash distribution from volcanic eruptions varied by distance from the crater: coarse ash was concentrated within 20 km, a mix of coarse and fine ash between 20-40 km, and fine ash predominated beyond 40 km. NIRD rates significantly increased between 1994 and 2003, correlating with the distribution and frequency of ash plumes, particularly the amount of fine particles smaller than 10 μm. Municipalities with frequent fine ash dispersion had higher NIRD rates. Chronic health effects persisted even during periods of decreased ash activity. Fine particles, especially those smaller than 2.5 μm, were identified as the primary factor affecting respiratory health, as they can reach deep into the respiratory system and cause lasting damage. |
| Yglesias-González M, et al. 2014 | Costa Rica | Turrialba | Erupting volcano | Gases (CO_2_, SO_2_) | 2010  Continuous activity | General population | Epidemiological study | The study found that children and elderly individuals are at the highest risk of developing throat discomfort. Female physiology, exposure to gases, and domestic activities contribute to their vulnerability. Outdoor workers have a higher risk (OR=1.51) of experiencing throat discomfort compared to indoor workers. Additionally, risk factors were elevated in communities exposed to the Turrialba volcano. |
| Carlsen HK, et al. 2012‡ | Iceland | Eyjafjallajökull | Erupting volcano | Ash (maximum deposit ~1000 g/m² near the vent; ~200 g/m² in Vík. Fine particle fraction: 25% <10 μm in the first days) | 2010 | General population | Epidemiological study | The study found that exposure to volcanic activity did not cause significant physical health impairments. No hospital admissions, fatalities, or serious symptoms were linked to the eruption. The population showed better lung function, likely due to a lower smoking prevalence, compared to the general population. Nine percent of participants reported mental distress, with higher GHQ-12 (General Health Questionnaire) scores linked to feelings of helplessness, fear, or being disturbed by eruption-related noises. Most participants experienced eye and upper airway symptoms during the ash fall, with individuals having obstructive lung disease being severely affected. |
| Buist S, et al. 1985 | United States | St. Helens | Erupting volcano | Ash (not reported) | 1980 | General population | Epidemiological study | The study found a transient decrease in FEV_1_ in comparison to an appropriate control group during the first year of exposure. This decline was followed by a gradual recovery as exposure levels decreased. |
| Grob E, et al. 2012 | Chile | Chaitén  Cordón Caulle  Llaima and Villarica | Erupting volcanoes | Ash (N/A)  Gases (e.g. CO_2_, H_2_S, HF, SO_2_, SiO_2_) | 2008  2011  Continuous activity | General population | Narrative literature review | The study found that somatic health effects in the population due to a volcanic eruption are determined by ash and gas emissions. While volcanic ash poses a low risk to health, it can be very harmful to individuals with chronic respiratory diseases. The chemicals released during volcanic eruptions react with O_2_, water, and sunlight, leading to air contamination known as “volcanic smog.” This smog, when it encounters water, generates acid rain. Inhalation of this volcanic mist irritates mucous membranes and airways, potentially affecting lung function. For example, inhalation of SO_2_ causes acute bronchoconstriction. |
| Zabert I, et al. 2019 | Argentina | Calbuco | Erupting volcano | Ash (not reported) | 2015 | General population | Epidemiological study | The study assessed the prevalence of wheezing, respiratory, and ocular symptoms. The results showed no significant difference in wheezing prevalence between cities with high and low exposure levels (30.2% vs. 31.0%). Overall, a high proportion of the exposed population experienced wheezing and respiratory and ocular symptoms, with prevalence rates higher than the national average. No significant differences were found between the two cities in terms of symptoms. |
| Stewart C, et al. 2022 | N/A | Various erupting and quiescent volcanoes | Various erupting and quiescent volcanoes | Ash (N/A)  Gases (e.g. HCl, H_2_O, CO_2_, HF, CO, SO_2_, H_2_S)  Metals (e.g. Cu, Pb, Cd, Se)) | Various erupting and quiescent volcanoes | General population | Narrative literature review | Advances in volcanic air pollution assessment include standardized ash characterization methods, community-based air quality monitoring networks, and the development of forecasting and risk assessment tools. However, challenges remain, such as the lack of monitoring of health effects from major eruptions and prolonged degassing, as well as limited longitudinal studies on chronic exposure. Addressing these gaps requires co-developing standardized protocols, supporting local research teams, and fostering collaborations between researchers and local partners for effective communication of health risks. |
| Maldonado F, et al. 2018 | Ecuador | Tungurahua | Erupting volcano | Ash (not reported) | 2007 | General population | Epidemiological study | Exposure to ash from the Tungurahua volcano is associated with respiratory symptoms, as well as radiographic lung abnormalities and spirometric changes. |
| Higuchi K, et al. 2012 | Japan | Sakurajima | Erupting volcano | Ash (not reported) | 1972-2001 | General population | Epidemiological study | Residents experienced high mortality from respiratory diseases, including lung cancer and COPD. The study observed consistently elevated mortality rates for lung cancer in both men and women, indicating long-term health effects from the volcanic activity. Additionally, the area had high cumulative exposure to ashfall, which may contribute to the increased risk of lung cancer in the population. |
| Gudmundsson G. 2010 | Iceland | Eyjafjallajökull | Erupting volcano | Ash (N/A) | 2010 | General population | Narrative literature review | Volcanic ash exposure causes acute respiratory symptoms such as asthma and bronchitis and exacerbates pre-existing lung and heart conditions. Health impacts depend on ash particle size, composition, and exposure levels. No long-term effects on lung function or confirmed cases of silicosis have been reported. |
| Ishigami A, et al. 2008 | Japan | Miyakejima | Erupting volcano | Gases (SO_2_) | 2000 | General population | Epidemiological study | The incidence of respiratory symptoms is strongly linked to ambient concentrations of SO_2_. In Miyakejima, respiratory symptoms were significantly associated with volcanic SO_2_ exposure, with more severe impacts observed as exposure levels increased. |
| Forbes L, et al. 2003 | United Kingdom | Soufrière Hills | Erupting volcano | Ash (not reported) | 1995 | Children | Epidemiological study | The study found that volcanic ash exposure on Montserrat negatively impacted children’s respiratory health. Heavily exposed children had significantly higher odds of wheezing and exercise-induced bronchoconstriction compared to those with low exposure. These results highlight the importance of limiting children’s exposure to volcanic ash and managing respiratory symptoms effectively. |
| Yano E, et al. 1986 | Japan | Sakurajima | Erupting volcano | Ash (not reported)  Gases (SO_2_) | 1981 (1972-2001) | Women (30-59 years) | Epidemiological study | The study found a low prevalence of chronic respiratory symptoms among women exposed to volcanic ash near Mt. Sakurajima, with no significant differences between high and low exposure areas. Factors such as the large size, brief duration of high-dust exposure, and biological inertness of volcanic ash likely explain the minimal respiratory impact. |
| Baxter P, et al. 1983 | United States | St. Helens | Erupting volcano | Ash (Yakima: 8 mm; Other áreas: 2–4 mm) | 1980 | General population | Epidemiological study | Following the eruption there was an increase in emergency room visits due to asthma and bronchitis, particularly in areas affected by ashfall. A study of 39 asthma and 44 bronchitis patients revealed that a history of asthma, and possibly bronchitis, were risk factors for developing respiratory problems during this period. The main exacerbating factor was the elevated levels of airborne total suspended particulates. Additionally, a study of 97 patients with chronic lung disease who did not visit the hospital showed that approximately one-third experienced worsening symptoms due to the ashfall. |
| Carlsen HK, et al. 2021 | Iceland | Holuhraun | Erupting volcano (largest fissure eruption in >200 years) | Gases (SO_2_, SO_4_^2-^) | 2014-15 | General population | Epidemiological study | The study found that exposure to the mature volcanic plume from the 2014–15 Holuhraun eruption in Iceland, which was not forecasted in public advisories, was associated with a 23% increase in health care utilization for respiratory diseases and a 19.3% rise in asthma medication dispensing in Reykjavík. The lack of warnings for the mature plume led to increased visits to primary care doctors and hospital emergency departments. |
| Shimizu Y, et al. 2007 | Japan | Asama | Erupting volcano | Ash (>100 g/m² (high ashfall area), 10–100 g/m² (middle ashfall area)) | 2004 | General population | Epidemiological study | Patients in areas with higher levels of ashfall (100 g/m²) experienced significantly worse asthma symptoms compared to control areas. Both mild and moderate asthma cases in the ashfall area were more severely affected, with a significant decrease in peak expiratory flow rates after the eruption. Asthma treatment scores also increased. Symptoms like wheezing, chest tightness, and cough were more prevalent in the ashfall area. The ash particles were primarily composed of silica (60%) and other minerals like aluminum. |
| Linhares D, et al. 2015 | Portugal | Furnas | Quiescent volcano (active fumarolic fields and soil degassing) | Gases (CO_2_) | 1630  Continuous (hydrothermal) activity | General population | Epidemiological study | The study found significant differences in soil CO_2_ flux between areas. The prevalence of restrictive respiratory defects and COPD was significantly higher in the study group compared to controls. After adjusting volcanogenic air pollution was linked to higher rates of respiratory restrictions and obstructions, with increased severity in COPD. Respiratory obstructions were also significantly associated with age, fatigue, and smoking status, with asthma, smoking, and aging identified as key risk factors for COPD severity. |
| Hlodversdottir H, et al. 2016 | Iceland | Eyjafjallajökull | Erupting volcano | Ash (not reported) | 2010 | General population | Epidemiological study | The study found that residents exposed to the Eyjafjallajökull volcanic eruption in Iceland experienced long-term health effects, especially those with higher levels of exposure. In 2013, several physical symptoms were more prevalent in the exposed group compared to 2010, including morning phlegm, skin rash/eczema, back pain, insomnia, and increased use of asthma medications. The exposed group had higher prevalence of respiratory symptoms such as wheezing and phlegm, with some symptoms related to the level of exposure. The results suggest that volcanic eruption exposure increases the risk of certain symptoms up to 3–4 years later. |
| Tam E, et al. 2016 | United States | Kilauea | Erupting volcano | PM_2.5_:  Gases (SO_2_) | 1980  Continuous activity | Children | Epidemiological study | The study found that chronic exposure to volcanic air pollution (vog) from Kilauea Volcano on Hawai'i Island is associated with respiratory symptoms, particularly increased cough, and potentially reduced lung function (as measured by FEV_1_/FVC). The analysis identified four distinct vog exposure zones with varying levels of SO_2_, PM_2.5_, and acidity. While asthma and bronchitis diagnoses were not strongly associated with vog exposure, increased coughing was linked to acidic vog, suggesting its impact on respiratory health. |
| Mannino DM, et al. 1996 | United States | Kilauea | Erupting volcano | Gases (SO_2_) | 1980  Continuous activity | General population | Epidemiological study | Asthma was more common in younger individuals, while COPD was more prevalent in older individuals. Asthma-related emergency visits were higher in areas with intermittent volcanic exposure, while COPD visits were more frequent during years of increased volcanic activity. Meteorological factors, particularly westward winds and low temperatures, were strongly associated with higher asthma-related visits to the emergency department. |
| Amaral A, et al. 2006 | Portugal (Furnas and Santa Maria) | Furnas | Quiescent volcano (active fumarolic fields and soil degassing) | Gases (CO_2_)  Metals (^222^Rn) | 1630  Continuous (hydrothermal) activity | General population | Epidemiological study | This study investigated cancer incidence in two populations in the Azores: Furnas, a volcanic island with extinct volcanism but ongoing secondary activity, and Santa Maria, a non-volcanic area with no activity for approximately 3 million years. Data from 1991–2001 showed overall higher cancer rates in Furnas, particularly for lip, oral cavity, pharynx, and breast cancers, possibly linked to chronic exposure to volcanic gases and aerosols containing trace metals, including indoor ^222^Rn. In contrast, Santa Maria had higher rates of digestive, respiratory, and intrathoracic cancers, mainly in males. Relative risk analysis highlighted potential environmental impacts of volcanic activity on specific cancer types in Furnas. |
| Kochi T, et al. 2017‡ | Japan | Oyama | Erupting volcano | Gases (SO_2_) | 2000 | General population | Epidemiological study | Residents exposed to SO_2_ found no significant declines in pulmonary function. SO_2_ concentrations decreased significantly over the study period, especially in high-exposure areas. However, respiratory and irritative symptoms, including cough, throat irritation, nasal issues, and eye discomfort, increased in a dose-dependent manner. The findings suggest that while SO_2_ exposure did not impair lung function, it was associated with increased respiratory and irritative symptoms. |
| Naumova N, et al. 2007 | Ecuador | Guagua Pichincha | Erupting volcano | Ash (not reported) | 2000 | Children | Epidemiological study | The study examined the effects of the Guagua Pichincha eruption on respiratory health in children in Quito, Ecuador. It found a notable increase in emergency visits for respiratory infections and asthma, especially in younger children, following the eruption. The findings highlight a clear connection between volcanic ash exposure and respiratory issues in pediatric populations. |
| Iwasawa S, et al. 2009 | Japan | Oyama | Erupting volcano | Gases (SO_2_) | 2000 | General population | Epidemiological study | The study assessed respiratory health, despite no significant decline in lung function, participants reported an increase in respiratory symptoms, such as cough and phlegm, in 2006 compared to 2004. The prevalence of chronic bronchitis-like symptoms also increased. Areas with higher SO_2_ concentrations showed more frequent respiratory issues, including phlegm and nasal irritation. These findings suggest that SO_2_ exposure contributed to the respiratory symptoms observed in the residents. |
| Zabert I, et al. 2020 | Argentina | Calbuco | Erupting volcano | Ash (not reported) | 2015 | Adolescents | Epidemiological study | The study assessed asthma risk in adolescents exposed to volcanic ash one year after the Calbuco eruption, comparing two cities with different levels of exposure: San Carlos de Bariloche and Cipolletti. The prevalence of asthma symptoms was 14% in Bariloche and 10% in Cipolletti, with no significant statistical difference. Greater ash exposure was reported in Bariloche compared to Cipolletti. Despite higher ash exposure in Bariloche, no significant clinical impact on respiratory health was observed in either population. |
| Choudhury AH, et al. 1997 | United States | Anchorage | Erupting volcano | PM_10_ | 1992 | General population | Epidemiological study | This study examined the relationship between daily morbidity and respirable PM_10_ in Anchorage, Alaska, using insurance claims data for state employees and their dependents. Medical visits for asthma, bronchitis, and upper respiratory infections were analyzed in relation to PM_10_ levels measured by air-monitoring sites. The regression analysis revealed a significant positive association between morbidity and PM_10_ pollution, with the strongest link observed on days with concurrent high PM_10_ levels, particularly on warmer days. |
| Longo B, 2009‡ | United States | Kilauea | Erupting volcano | Gases (CO_2_) | 1983  Continuous activity (until 2008) | General population | Epidemiological study | There were statistically significant increased odds associated with exposure for self-reported cough, phlegm, rhinorrhea, sore and dry throat, sinus congestion, wheezing, eye irritation, and diagnosed bronchitis. |
| Longo B, et al. 2008† | United States | Kilauea | Erupting volcano | Gases (SO_2_) | 1983  Continuous activity (until 2008) | General population | Epidemiological study | Group analyses found no differences in pulse rate or BP; however, significantly faster mean pulse rates were detected in exposed non-medicated, non-smoking participants with BMI <25, and in participants aged > or = 65 years. Higher mean systolic BP was found in exposed participants with BMI <25. |
| Longo B, et al. 2010 | United States | Kilauea | Erupting volcano | PM_2.5_:  Gases (SO_2_) | 1983  Continuous activity (until 2008) | General population | Epidemiological study | The study found a significant increase in medically diagnosed acute illnesses during a period of high sulfurous volcanic air pollution (vog) from the Kilauea Volcano in 2008. There were strong associations between high vog exposure and increased visits for conditions such as cough, headache, acute pharyngitis, and acute airway problems, with a six-fold increase in odds for acute airway problems, particularly among young Pacific Islanders. These findings suggest that elevated volcanic emissions are linked to increased morbidity in certain subgroups of the population. |
| Longo B, et al. 2008 | United States | Kilauea | Erupting volcano | Gases (SO_2_) | 1983  Continuous activity (until 2008) | General population | Epidemiological study | The study found that communities exposed to sulfurous volcanic air pollution from Kilauea Volcano had a significantly higher risk of acute bronchitis. The incidence of acute bronchitis was higher in exposed communities, with the greatest risk observed in children and middle-aged females. These findings suggest that continuous exposure to volcanic air pollution increases the risk of acute bronchitis. |
| Yano E, et al. 1990 | Japan | Sakurajima | Erupting volcano | Ash (not reported)  Gases (SO_2_) | 1985-6 (1972-2001) | General population | Epidemiological study | The prevalence of chronic bronchitis and other respiratory symptoms was low across the areas, indicating that volcanic ash was not a major cause of respiratory disease overall. The increased prevalence of symptoms in higher ash exposure areas suggests a mild association between volcanic ash and respiratory issues. |
| Bergin C, et al. 2021 | New Zealand | Whakaari/White Island | Erupting volcano | Pyroclastic flow | 2019 | General population | Epidemiological study | This study describes the pulmonary effects of exposure to a volcanic pyroclastic flow. The patients, who were close to the eruption's fissure, underwent chest radiographs, CT scans, and bronchoscopy. Findings showed a range of lung injuries, including ARDS in two patients, which improved within three months. Most patients had chest imaging abnormalities on admission, and eight required ongoing ventilation. The study concludes that the lung abnormalities were likely due to toxic gas emissions from the eruption. |
| Kraemer M, et al. 1985 | United States | St. Helens | Erupting volcano | Gases (SO_2_) | 1980  Continuous activity (until 2008) | Children | Epidemiological study | Spokane County saw a nearly twofold increase in pediatric asthma hospitalizations during weeks of heavy volcanic ash exposure (>1000 µg/m³). This rise, absent significant viral outbreaks, suggests volcanic ash acted as a respiratory irritant, exacerbating asthma in a small subset of children (<1%). Over hospitalization due to fear of ash exposure and limited transportation during the eruption may have influenced the data, but the findings highlight the potential bronchospastic effects of extreme particulate exposure. |
| Baxter P, et al. 198 | United States | St. Helens | Erupting volcano | Ash (N/A)  Gases (CO_2_, SO_2_, HCl, HF, CO, H_2_S)  Pyroclastic and mud flows | 1980  Continuous activity (until 2008) | General population | Narrative literature review | Volcanic eruptions pose multiple hazards: pyroclastic flows and lahars cause rapid destruction, burns, and fatalities; ashfall exacerbates respiratory conditions, particularly in vulnerable populations; and toxic gases like SO₂ and CO₂ threaten health and ecosystems. Maintaining distance from craters and improving shelter and healthcare access can mitigate these risks. |
| Horwell CJ, et al. 2006 | N/A | Various erupting volcanoes | Various erupting volcanoes | Ash (N/A) | Various erupting volcanoes | General population | Systematic review | Volcanic ash particles pose respiratory health risks influenced by their size, shape, and mineral composition. Acute exposure can exacerbate asthma, bronchitis, and pre-existing lung conditions, while chronic exposure may lead to silicosis, COPD, and pneumoconiosis. Historical eruptions, such as Irazu and Mt. St. Helens, revealed acute and chronic respiratory impacts, with varying toxicity based on ash composition. Notably, cristobalite-rich ash, like that from Soufrière Hills, presents significant silicosis risks. Mixed findings from global volcano studies emphasize the need for ongoing monitoring, hazard mitigation, and research to understand long-term health effects and variations in ash toxicity. |
| Carlsen HK, et al. 2012‡ | Iceland | Eyjafjallajökull | Erupting volcano | Ash (not reported in thickness units; ash deposits described as ~1000 g/m² near the volcano; ~200 g/m² at 50 km (Vík)) | 2010 | General population | Epidemiological study | The exposed population showed a significantly higher prevalence of respiratory symptoms, including chest tightness, cough, and chronic bronchitis, with odds ratios ranging from 1.9 to 2.6. These symptoms increased with ash exposure levels, particularly in high-exposure areas where risks of chest tightness, chronic phlegm, and recent symptoms like cough and phlegm were notably higher. Nasal, eye, and upper respiratory symptoms were also more frequent in the exposed group, increasing proportionally with exposure level, and women were more affected. |
| Fano V, et al. 2010 | Italy | Etna | Erupting volcano | PM_10_:  Ash (not reported)  Gases (SO_2_) | 2002 | Adult patients with asthma | Epidemiological study | The 2002 eruption of Mt. Etna caused significant environmental and health impacts. PM_10_ levels increased by 284% and sulfur dioxide by 385% compared to the previous year, with particulate levels correlating with seismic activity. Public health measures were implemented, including school closures and protective equipment distribution. Mortality due to respiratory diseases decreased during the eruption, while hospital admissions for cardiovascular diseases, particularly ischemic and cerebrovascular conditions, increased, especially in individuals over 65. Admissions for eye disorders doubled, although not statistically significant. Time-series analysis suggested delayed cardiovascular effects linked to PM_10_ exposure. |
| Hlodversdottir H, et al. 2018 | Iceland | Eyjafjallajökull | Erupting volcano | Ash (not reported) | 2010 | Children | Epidemiological study | Children exposed to the 2010 Eyjafjallajökull eruption experienced increased respiratory symptoms and anxiety compared to non-exposed children, with boys also reporting more headaches and sleep disturbances. Children from damaged homes faced higher risks of anxiety and depressed mood. Symptoms persisted up to three years post-eruption. |
| Elliot AJ, et al. 2010 | Iceland | Eyjafjallajökull | Erupting volcano | Ash (not reported) | 2010 | General population | Epidemiological study | The study found that increases in respiratory symptoms during the volcanic ash incident were likely due to seasonal factors, such as higher pollen levels, rather than the eruption itself. Environmental monitoring showed minimal exposure to ash, and syndromic surveillance revealed no abnormal health patterns. Overall, the health impact of the ash cloud was minimal, and real-time health data provided reassurance to the public. |
| Oudin A, et al. 2013 | Iceland | Grímsvötn | Erupting volcano | Ash (not reported) | 2011 | General population | Epidemiological study | The study investigated the potential impact of the volcanic eruption on mortality. Data on daily all-cause mortality were analyzed from areas exposed to ash and unexposed areas. No absolute increase in mortality was observed during the exposure period (24 May–31 May), but the mean mortality ratio was slightly higher in the Ash area compared to the No ash area. However, the differences were not statistically significant, and the statistical power was low due to the short exposure period. |
| Carlsen HK, et al. 2019 | Iceland | Holuhraun | Erupting volcano | Gases (SO_2_) | 2014-2015 | Workers | Epidemiological study | This study evaluated the impact of SO_2_ exposure on individuals working at a volcanic eruption site. It found that while lung function remained normal before and after exposure, participants reported mild, temporary respiratory symptoms such as eye and nasal irritation during exposure. However, there were no signs of significant airway inflammation or obstruction. The study suggests that SO_2_ exposure caused mild symptoms, though “healthy-worker” effects could influence the results. |
| Iwasawa S, et al. 2015 | Japan | Miyakejima | Erupting volcano | Gases (SO_2_) | 2000  Continuous volcanic activity | Children | Epidemiological study | This study assessed the health effects of SO_2_ exposure on children. SO_2_ concentrations decreased over the years, with higher concentrations in more exposed areas. The results showed that children in highly exposed areas had higher rates of symptoms like throat and eye irritation, with a threshold concentration of approximately 30 ppb. However, spirometry tests did not reveal significant differences in lung function. The study concluded that SO_2_ exposure was associated with increased irritative symptoms in children, though it did not affect pulmonary function. |
| Shiozawa M, et al. 2018 | Japan | Miyakejima | Erupting volcano | Gases (SO_2_) | 2000  Continuous volcanic activity | General population | Epidemiological study | The study found that exposure to SO_2_ from the Miyakejima volcano caused respiratory and irritative symptoms in residents, particularly among the younger population due to greater exposure from outdoor work. Although lung functions were not affected, some symptoms were more common in areas with higher SO_2_ concentrations. Displaced inhabitants reported increased levels of anxiety and insomnia. It was concluded that prolonged exposure to volcanic gas impacted both physical and mental health. |
| Rojas-Ramos M, et al. 2001 | Mexico | Popocatepetl | Erupting volcano | Ash (not reported) | 1994  Continuous volcanic activity | Non-smoking farmers | Epidemiological study | This study followed non-smoking farmers exposed to volcanic ash. Spirometry showed reduced lung function during the exposure period, which returned to normal after seven months. Respiratory and ocular symptoms also improved. These results suggest short-term volcanic ash exposure causes reversible airway inflammation, likely due to the low volume and non-fibrogenic nature of the ash. |
| Bates MN, et al. 2015 | New Zealand | Rotorua | Quiescent volcano | Gases (H_2_S) | Continuous (geothermal) emissions | General population | Epidemiological study | This study found that the median H_2_S concentration in participants' residences was 20.3 ppb, and in workplaces, it was 26.4 ppb. Analysis of long-term H_2_S exposure did not show a clear association with spirometric parameters or COPD. However, current H_2_S exposure indicated better lung function in higher exposure quartiles compared to the lowest. No significant patterns were found for asthma or COPD in relation to H_2_S exposure, though smoking, age, and ethnicity were significant factors in respiratory health. |
| Bates MN, et al. 2013 | New Zealand | Rotorua | Quiescent volcano | Gases (H_2_S) | Continuous (geothermal) emissions | General population | Epidemiological study | This study investigated the potential link between long-term low-level H_2_S exposure and asthma, where ambient H_2_S levels are relatively high due to geothermal sources. The results showed no clear evidence of increased asthma risk from H_2_S exposure. However, a reduced risk of diagnosed asthma and asthma symptoms, particularly wheezing, was observed in areas with higher H_2_S exposure. This pattern aligns with evidence suggesting that H_2_S may have signaling functions in the body, including promoting smooth muscle relaxation and reducing inflammation. |
| Durand M, et al. 2006 | New Zealand | Rotorua | Quiescent volcano | Gases (H_2_S) | Continuous (geothermal) emissions | General population | Epidemiological study | This study found that residents living near the geothermal field in Rotorua, New Zealand, may have a higher risk of non-infectious respiratory diseases, particularly COPD, due to chronic low-level exposure to H_2_S. Spatial analysis of hospital discharge data revealed disease clusters in areas with higher H_2_S emissions. |
| Bernstein RS, et al. 1986 | United States | St. Helens | Erupting volcano | Ash (not reported)  Gases (SO_2_)  Pyroclastic and mud flows | 1980  Continuous activity (until 2008) | General population | Epidemiological study | Surveillance and observational studies indicated that: excess in morbidity were limited to transient increases in ER visits and hospital admissions for traumatic injuries and respiratory problems (but not for communicable disease or mental health problems) which were associated in time, place, and person with exposures to volcanic ash; excessive mortality due to suffocation (76%), thermal injuries (12%), or trauma (12%) by ash and other volcanic hazards was directly proportional to the degree of environmental damage–that is, it was more pronounced among those persons (48/65, or about 74%) who, at the time of the eruption, were residing, camping, or sightseeing (despite restrictions) or working (with permission) closer to the crater in areas affected by the explosive blast, pyroclastic and mud flows, and heavy ashfall; and de novo appearance of ash-related asthma was not observed, but transient excesses in adverse respiratory effects occurred in two high-risk groups–hyper-susceptible (with preexisting asthma or chronic bronchitis) and heavily exposed workers. |

**Abbreviations:** *^222^Rn*: radon; *ARDS*: acute respiratory distress; *BP*: blood pressure; *BMI*: body mass index; *Cd:* cadmium; *CO*: carbon monoxide; *CO_2_*: carbon dioxide; *COPD*: chronic obstructive pulmonary disease; *CT*: computed tomography; *Cu*: copper; *ED*: emergency department; *ER:* emergency room; *FVC:* forced vital capacity; *FEV*: forced expiratory volume; *H_2_O*: water; *H_2_S*: hydrogen sulfide; *HCl*: hydrochloric acid; *HF*: hydrofluoric acid; *IHD*: ischemic heart disease; *N/A:* not applicable; *NIRD*: non-infectious respiratory disease; *OR*: odds ratio; *Pb*: lead; *PM*: particulate matter; *PPB:* parts per billion; *PPM:* parts per million; *RR:* relative risk; *Se*: selenium; *SiO_2_*: silicon dioxide; *SO_2_*: sulfur dioxide; *SO_4_^2-^*: sulfate ion †: included by PICO of cardiovascular diseases; ‡: included by PICO of ocular diseases.

### Supplementary Table 2b: Data extraction and synthesis of findings on cardiovascular diseases (n=5).

| **Author, Year** | **Country** | **Volcano** | **Type of activity** | **Volcanic product (dimension)** | **Year of eruption** | **Population** | **Study design** | **Findings** |
| --- | --- | --- | --- | --- | --- | --- | --- | --- |
| Lombardo D, et al. 2013 | Italy | Etna | Erupting volcano | Ash (not reported) | 2002 | General population | Epidemiological study | Significant increase of ED visits for acute respiratory and cardiovascular diseases, and ocular disturbances during the ash exposure period. |
| Longo B, et al. 2008 | United States | Kilauea | Erupting volcano | Gases (SO_2_) | 1983  Continuous activity (until 2008) | General population | Epidemiological study | Group analyses found no differences in pulse rate or BP; however, significantly faster mean pulse rates were detected in exposed non-medicated, non-smoking participants with BMI <25, and in participants aged > or = 65 years. Higher mean systolic BP was found in exposed participants with BMI <25. |
| Hickling J, et al. 1999 | New Zealand | Ruapehu | Erupting volcano | Ash (>0.25 mm) | 1996 | General population | Epidemiological study | The only detrimental effects detected were a borderline increase in acute bronchitis (RR=1.44, p=0.032) and conflicting evidence for some cardiovascular diseases. |
| Fano V, et al. 2005 | Italy | Etna | Erupting volcano | PM_10_ | 2002 | General population | Epidemiological study | No excess mortality was observed during eruption periods. However, an increase in hospitalizations for circulatory system diseases was observed, as well as for ischemic diseases (RR 1.31; 95% CI: 1.10-1.56), myocardial infarction (RR 1.34; 95% CI: 1.02-1.76) and cerebrovascular diseases (RR 1.24; 95% CI: 1.05-1.47). The excess was more marked among the elderly. |
| Carapezza ML, et al. 2023 | Italy | Colli Albani | Quiescent volcano | Gases (SO_2_, CO_2_) | 20,000-23,000 years ago  Continuous (degassing) activity | General population | Epidemiological study | The study found increased cardiovascular risks near volcanic gas emissions. Men in zone A had higher mortality from cardiovascular disease (HR 1.60; 95% CI: 0.95–2.70) and myocardial infarction (HR 2.11; 95% CI: 0.91–4.90), while women in zones A+B showed elevated mortality from ischemic heart disease (HR 1.51; 95% CI: 0.95–2.41). Women in zone A had markedly higher ED visits for precordial pain (HR 3.79; 95% CI: 1.27–11.30) and rhythm disturbances (HR 1.17; 95% CI: 1.09–1.26 in zone B; HR 1.12; 95% CI: 1.05–1.20 in zones A+B). |

**Abbreviations:** *BMI*: body mass index; *BP*: blood pressure; *CO_2_*: carbon dioxide*; CI*: confidence interval; *ED*: emergency department; *HR:* hazard ratio; *N/A*: not applicable; *PM*: particulate matter; *RR*: relative risk; *SO_2_*: sulfur dioxide.

### Supplementary Table 2c: Data extraction and synthesis of findings on ocular diseases (n=11).

| **Author, Year** | **Country** | **Volcano** | **State of activity** | **Volcanic product (dimension)** | **Year of eruption** | **Population** | **Study design** | **Findings** |
| --- | --- | --- | --- | --- | --- | --- | --- | --- |
| Longo B, 2009 | United States | Kilauea | Erupting volcano | Gases (CO_2_) | 1983  Continuous activity (until 2008) | General population | Epidemiological study | There were statistically significant increased odds associated with exposure for self-reported cough, phlegm, rhinorrhea, sore and dry throat, sinus congestion, wheezing, eye irritation, and diagnosed bronchitis. |
| Kochi T, et al. 2017 | Japan | Miyakejima | Erupting volcano | Gases (SO_2_) | 2000 | General population | Epidemiological study | Prevalence of subjective symptoms such as “cough,” “irritation and/or pain in throat,” “irritation, runny nose, and/or nasal sniffles,” and “irritation and/or pain in the eyes,” dependently increased on SO_2_ concentration. |
| Carlsen HK, et al. 2012 | Iceland | Eyjafjallajökull | Erupting volcano | Ash (not reported in thickness units; ash deposits described as ~1000 g/m² near the volcano; ~200 g/m² at 50 km (Vík)) | 2010 | General population | Epidemiological study | The likelihood of having symptoms during the last month was higher in the exposed population, such as; tightness in the chest (OR 2.5; 95% CI: 1.1 to 5.8), cough (OR 2.6; 95% CI: 1.7 to 3.9), phlegm (OR 2.1; 95% CI: 1.3 to 3.2), eye irritation (OR 2.9; 95% CI: 2.0 to 4.1) and psychological morbidity symptoms (OR 1.3; 95% CI: 1.0 to 1.7). |
| Carlsen HK, et al. 2012 | Iceland | Eyjafjallajökull | Erupting volcano | Ash (maximum deposit ~1000 g/m² near the vent; ~200 g/m² in Vík. Fine particle fraction: 25% <10 μm in the first days) | 2010 | General population | Epidemiological study | Almost half of the adult participants (43%) experienced symptoms from the upper airways and eyes during the ash fall. Almost everybody or 153 of 161 (96%) found facial masks and glasses protective against respiratory and eye symptoms when staying outdoors during ash fall. |
| Camara JG, et al. 2011 | United States | Kilauea | Erupting volcano | Ash (not reported)  Gases (SO_2_) | 1983  Continuous activity | General population | Epidemiological study | Ocular signs noted were conjunctival injection (100%), clear mucous discharge (100%), papillary reaction (100%), punctual edema (80%), eyelid swelling (73.3%) and chemosis (63.3%). Ocular symptoms were itchiness (100%), foreign body sensation (100%), tearing (96.6%) and burning sensation (90%). |
| Ortiz E, et al. 2022 | Costa Rica | Poás  Turrialba | Erupting volcanoes | Gases (SO_2_, H_2_S, HCl, HNO_3_, HF) | 2017  2014 | Workers (National System of Conservation Areas) | Epidemiological study | The principal results (pollutants above the detection limit) reported for the two national parks indicated that the highest value in this study was that of SO_2_ (average of 3 h) in the Poás volcano (1.90 ± 0.11) mg/m^3^. The highest concentration of SO_2_ in Turrialba was (0.40 ± 0.04) mg/m^3^ during the same period. During the evaluation of park rangers’ health, it was found that the most frequently reported pathologies that impact health in the study population were irritation of the mucous membrane eyes (n=9), headaches (n=8), and fatigue (n=8). |
| Kimura K, et al. 2005 | Japan | Sakurajima | Erupting volcano | Ash (average of 6457.1 g/m²/year (high exposure, 4 km from the crater) vs. 301.2 g/m²/year (low exposure, 10 km from the crater)) | 2000 | Children | Epidemiological study | Subjects in the high-exposure area showed ocular symptoms more often than those in the low-exposure area (p<0.0001). Years of active volcanic eruptions (volcanic ash of 5000 g/m^2^/year or more) were closely related to years with a high frequency of ocular symptoms in subjects in the high-exposure area (p<0.05) but related conversely in subjects in the low-exposure area (p<0.01). Major ocular symptoms were redness, discharge, foreign body sensation, and itching, all treated effectively with eye drops. |
| Fraunfelder FT, et al. 1983 | United States | St. Helens | Erupting volcano | Ash (not reported) | 1980  Continuous activity (until 2008) | General population | Epidemiological study | Although the ash particles acted as ocular foreign bodies, the small particles were apparently well tolerated for the most part, except for acute irritation. Patients with contact lenses or sicca syndrome had the most frequent ocular complaints. |
| Gudmundsson G, et al. 2016 | Iceland | Skaftáreldar  Eyjafjallajökull  Grímsvötn  Holuhraun | Erupting volcanoes | Ash (N/A)  Gases (SO_2_, HF, HCl, CO_2_, H_2_O vapor)  Aerosols (H_2_SO_4_) | 1783-4  2010  2011  2014-5 | General population | Narrative literature review | Volcanic gases expelled during eruptions can be highly toxic for humans if their concentrations are high, irritating the mucus membranes of the eyes and upper respiratory tract at lower concentrations. They can also be very irritating to the skin. Volcanic ash is also irritating for the mucus membranes of the eyes and upper respiratory tract. The smallest particles of volcanic ash can reach the alveoli of the lungs. |
| Cook A, et al. 2011 | N/A | Various erupting and quiescent volcanoes | Various erupting and quiescent volcanoes | Ash (N/A)  Gases (SO_2_, H_2_S, CO, CO_2_, H_2_O, HF)  Pyroclastic and mud flows | Various erupting and quiescent volcanoes | General population | Narrative literature review | Volcanic and geothermal events are among the most dramatic of all natural phenomena, and they pose numerous risks to human health. Volcanic vents and fissures provide a conduit by which magma - the molten rock, gases, and water within the earth - may disperse and cause illness in exposed communities. The majority of casualties due to volcanic activity in the past few centuries are result of pyroclastic flows, lahars, and suffocation or building collapse from ash or debris; tsunamis, which may spread for hundreds of miles; and indirect consequences of eruptions, such as famine or infectious disease outbreaks. As with many other disasters, population displacement secondary to volcanic emergencies creates attendant problems of poor sanitation, overcrowding, and contamination of food or water sources. Apart from the thermal and physical injuries resulting from an eruption, ejecta may also contain toxic elements and compounds, including silica, fluoride, and heavy metals, which may lead to risks of acute or chronic toxicity. These compounds may be carried within eruptive columns, plumes, or runoff and thus have health impacts at a significant distance from the active site. |
| Grattan J, et al. 2003 | Iceland | Skaftáreldar | Erupting volcano | Gases (SO_2,_ H_2_S, F(p)) | 1783-4 | General population | Narrative literature review | It is noted that many common symptoms of severe exposure to air pollution can be linked to the dry fog of 1783; these included difficulty in breathing, eye and skin irritation, headaches, loss of appetite and tiredness. |

**Abbreviations:** *CI*: confidence interval; *CO*: carbon monoxide; *CO_2_*: carbon dioxide; *F*: fluorine; *H_2_O*: water; *H_2_S*: hydrogen sulfide; *H_2_SO_4_*: sulfuric acid; *HCl*: hydrochloric acid; *HF*: hydrofluoric acid; *HNO_3_*: nitric acid; *N/A:* not applicable*; OR*: relative risk; *SO_2_*: sulfur dioxide.

### Supplementary Table 2d: Data extraction and synthesis of findings on dermatological diseases (n=11).

| **Author, Year** | **Country** | **Volcano** | **State of activity** | **Volcanic product (dimension)** | **Year of eruption** | **Population** | **Study design** | **Findings** |
| --- | --- | --- | --- | --- | --- | --- | --- | --- |
| Lombardo D, et al. 2013^†^ | Italy | Etna | Erupting volcano | Ash (not reported) | 2002 | General population | Epidemiological study | Significant increase of ED visits for acute respiratory and cardiovascular diseases, and ocular disturbances during the ash exposure period. |
| Hlodversdottir H, et al. 2016^¥^ | Iceland | Eyjafjallajökull | Erupting volcano | Ash (not reported) | 2010 | General population | Epidemiological study | In the exposed group, certain symptoms were higher in 2013 than in 2010, for example, morning phlegm during winter (OR 2.14; 95% CI: 1.49 to 3.06), skin rash/eczema (OR 2.86; 95% CI: 1.76 to 4.65), back pain (OR 1.45; 95% CI: 1.03 to 2.05) and insomnia (OR 1.53; 95% CI: 1.01 to 2.30), in addition to a higher prevalence of regular use of certain medications (e.g., for asthma (OR 2.80; 95% CI: 1.01 to 7.77)). |
| Gudmundsson G, et al. 2016^‡^ | Iceland | Skaftáreldar  Eyjafjallajökull  Grímsvötn  Holuhraun | Erupting volcanoes | Ash (N/A)  Gases (SO_2_, HF, HCl, CO_2_, H_2_O vapor)  Aerosols (H_2_SO_4_) | 1783-4  2010  2011  2014-5 | General population | Narrative literature review | Volcanic gases expelled during eruptions can be highly toxic for humans if their concentrations are high, irritating the mucus membranes of the eyes and upper respiratory tract at lower concentrations. They can also be very irritating to the skin. Volcanic ash is also irritating for the mucus membranes of the eyes and upper respiratory tract. The smallest particles of volcanic ash can reach the alveoli of the lungs. |
| Baker P, et al. 2022 | New Zealand | Whakaari/White Island | Erupting volcano | Pyroclastic flows | 2019 | General population | Epidemiological study | Thirty-one entered into the New Zealand National Burn Service across four hospitals. The median age of the patients treated at the National Burn Centre was 45.5 years (range: 14-67 years) and median TBSA burn was 49.5% (range: 9%-90%). The 3-month survival of this eruptive event was 55%, which subsequently fell to an overall rate of 53% following one late death of an early survivor after repatriation home. Of the patients who survived the initial eruption for long enough to be admitted to the National Burn Service, the overall survival rate was 71% at 3 months. We describe 12 lessons we have learnt from our management of the survivors. The key surgical lessons among these are: 1) The injuring mechanism combined ballistic trauma, thermal and acidic burn components, with the acid component being the most problematic and urgent for management; 2) Volcanic ash burns result in ongoing burn depth progression, deep underlying tissue damage and significant metabolic instability; 3) Early skin grafting was not successful in many cases; 4) Reconstructive strategy needed adjusting to cope with the high operative demand and limited donor sites in all patients; 5) Protect yourself from potential dangers with additional personal protective equipment in an unfamiliar setting. |
| Baxter PJ, et al. 1989 | Cameroon | Lake Nyos | Erupting volcano (sudden limnic eruption from volcanic crater lake) | Gases (CO_2_) | 1986 | General population | Epidemiological study | Rescuers noted cutaneous erythema and bullae on an unknown proportion of corpses and 161 (19%) survivors treated in hospital; though these lesions were initially believed to be burns from acidic gases, further investigation suggested that they were associated with coma states caused by exposure to carbon dioxide in air. |
| Baxter PJ, et al. 2017 | Indonesia | Merapi | Erupting volcano | Pyroclastic flows | 1986  2010 | General population | Epidemiological study | In our series, 106 patients from the two eruptions were treated in the same major hospital in Yogyakarta and a third of these survived. Seventy-eight per cent were admitted with over 40% TBSA burns and around 80% of patients were suspected of having at least some degree of inhalation injury as well. Thirty-five patients suffered over 80% TBSA burns and only one of these survived. Crucially, 45% of patients were in the 40-79% TBSA range, with most suspected of suffering from inhalation injury, for whom survival was most dependent on the hospital treatment they received. |
| Beylin D, et al. 2022 | N/A | Various erupting and quiescent volcanoes | Various erupting and quiescent volcanoes | Ash (N/A)  Gases (SO_2_, CO_2_)  Pyroclastic and mud flows  Sulfate aerosols | Various erupting and quiescent volcanoes | General population | Systematic review | Injuries acquired, and health impacts were categorized by onset-direct/indirect or immediate/delayed. Health concerns following an eruption were categorized: (1) respiratory; (2) ocular; and (3) skin, including deep tissues. |
| Locke M, et al. 2021 | New Zealand | Whakaari/White Island | Erupting volcano | Pyroclastic flows | 2019 | General population | Epidemiological study | Results show that the average variance for operative minutes was significantly above predicted with both the full-thickness burn model (average variance 3.24) and the electrical burn model (average variance 2.65). There was a wide range in both cases (0.54-6.17 and 0.44-5.06, respectively). There was less variance from predicted values of operative visits required than operative minutes (mean: 1.58; range 0.9-3.02). Overall, the values for patients with smaller burns showed the greatest variability from predictions with regard to the total number of operative visits during the first 4 weeks of care. |
| Parshley PF, et al. 1982 | United States | St. Helens | Erupting volcano | Pyroclastic flows | 1980  Continuous activity (until 2008) | General population | Case series | Evidence of inhaled ash complicating various stages of adult respiratory distress syndrome was confirmed by energy dispersive roentgenographic analysis. In the Pacific Northwest, Alaska, and the Aleutian Islands, potential for further injuries of this type in even larger numbers exists. Should these occur, those who treat the victims should be aware of the potential for severe inhalation problems in addition to the obvious burns. |
| Russo M, et al. 2015 | Italy | Etna | Erupting volcano | Not described because the study aim | 2002 | General population | Epidemiological study | Considering 72,197 incident cases, thyroid cancer (IRR=1.68 in females and 1.40 in males) and lymphatic leukemia (IRR: females=1.48, males=1.39) were significantly increased in the volcanic area in both men and women. Hodgkin's lymphoma, stomach and breast cancer in women and prostate cancer in men were also significantly increased in the volcanic area. |
| Wakisaka I, et al. 1989 | Japan | Sakurajima | Erupting volcano | Gases (SO_2_) | 1981 (1972-2001) | General population | Epidemiological study | Few or no stable seasonalities were shown in the variations of monthly clinical consultations for patients with the other diseases studied, rhinitis, other respiratory diseases and conjunctivitis. In addition, the variations in the monthly averages of SO_2_ concentrations showed a stable seasonality with the highest peak in winter and the lowest in summer but there was no stable seasonality in the monthly variations of total suspended particles at the place in Arimura for which air pollution data were available. These facts suggest that in the area exposed to volcanic air pollution, the seasonal variation in the number of monthly clinical consultations for respiratory problems is partly modified by the exposure levels of SO_2_ rather than total suspended particles, although no adjustments were made for climatologic factors. |

**Abbreviations:** *CO_2_*: carbon dioxide; *CI*: confidence interval; *ED*: emergency department; *H_2_O*: water; *H_2_SO_4_*: sulfuric acid; *HCl*: hydrochloric acid; *HF*: hydrofluoric acid; *IRR*: incident rate ratio; *OR*: odds ratio; *N/A*: not applicable; *SO_2_*: sulfur dioxide*; TBSA*: total body surface area. †: included by PICO of cardiovascular diseases; ¥ included by PICO of respiratory diseases; ‡ included by PICO of ocular diseases.

### Supplementary Table 2e: Data extraction and synthesis of findings on mental health diseases (n=20).

| **Author, Year** | **Country** | **Volcano** | **Type of activity** | **Volcanic product (dimension)** | **Year of eruption** | **Population** | **Study design** | **Findings** |
| --- | --- | --- | --- | --- | --- | --- | --- | --- |
| Carlsen HK, et al. 2012^‡^ | Iceland | Eyjafjallajökull | Erupting volcano | Ash (not reported in thickness units; ash deposits described as ~1000 g/m² near the volcano; ~200 g/m² at 50 km (Vík)) | 2010 | General population | Epidemiological study | The likelihood of having symptoms during the last month was higher in the exposed population, such as; tightness in the chest (OR 2.5; 95% CI: 1.1 to 5.8), cough (OR 2.6; 95% CI: 1.7 to 3.9), phlegm (OR 2.1; 95% CI: 1.3 to 3.2), eye irritation (OR 2.9; 95% CI: 2.0 to 4.1) and psychological morbidity symptoms (OR 1.3; 95% CI: 1.0 to 1.7). |
| Hlodversdottir H, et al. 2016^¥^ | Iceland | Eyjafjallajökull | Erupting volcano | Ash (not reported) | 2010 | General population | Epidemiological study | In the exposed group, certain symptoms were higher in 2013 than in 2010, for example, morning phlegm during winter (OR 2.14; 95% CI: 1.49 to 3.06), skin rash/eczema (OR 2.86; 95% CI: 1.76 to 4.65), back pain (OR 1.45; 95% CI: 1.03 to 2.05) and insomnia (OR 1.53; 95% CI: 1.01 to 2.30), in addition to a higher prevalence of regular use of certain medications (e.g., for asthma (OR 2.80; 95% CI: 1.01 to 7.77)). |
| Hlodversdottir H, et al. 2018^¥^ | Iceland | Eyjafjallajökull | Erupting volcano | Ash (not reported) | 2010 | Children | Epidemiological study | In 2010, exposed children were more likely than non-exposed children to experience respiratory symptoms (medium exposed OR 1.47; 95% CI: 1.07–2.03; high exposed OR 1.52; 95% CI: 1.03–2.24) and anxiety/worries (medium exposed OR 2.39; 95% CI: 1.67–3.45; high exposed OR 2.77; 95% CI: 1.81–4.27). Both genders had an increased risk of symptoms of anxiety/worries but only exposed boys were at increased risk of experiencing headaches and sleep disturbances compared to non-exposed boys. Within the exposed group, children whose homes were damaged were at increased risk of experiencing anxiety/worries (OR 1.62; 95% CI: 1.13–2.32) and depressed mood (OR 1.55; 95% CI: 1.07–2.24) than children whose homes were not damaged. Among exposed children, no significant decrease of symptoms was detected between 2010 and 2013. |
| Carlsen HK, et al. 2012^‡^ | Iceland | Eyjafjallajökull | Erupting volcano | Ash (maximum deposit ~1000 g/m² near the vent; ~200 g/m² in Vík. Fine particle fraction: 25% <10 μm in the first days) | 2010 | General population | Epidemiological study | Every other adult participant reported irritation in eyes and upper airway when exposed to volcanic ash. Adults (n=26) and children (n=5) with pre-existing asthma frequently reported worsening of their symptoms. No serious health problems requiring hospitalization could be attributed to the eruption. The majority of the participants reported no abnormal physical or mental symptoms to the examining physician. Compared to an age- and gender-matched reference group, the ash-exposed participants reported lower smoking rates and were less likely to have ventilation impairment. Less than 10% of the participants reported symptoms of stress, anxiety or depression. |
| Araki K, et al. 1998 | Japan | Unzen | Erupting volcano | Volcanic activity in general | 1991-5 | General population | Epidemiological study | One-quarter of the residents (about 52,000) in the disaster area around the volcanic eruption area of Mt. Unzen-Fugen have been forced to evacuate over a 3-year period. We conducted a psychological controlled study using the GHQ-30 (General Health Questionnaire) for evacuees. As a result, the percentage of people with a high score over 8 points was 67% of evacuees, markedly higher than 10% of the control group. Some mental support activities, such as counseling by community nurses and crisis intervention/medication by psychiatrists and others, have been promoted. Subjects who received a psychiatric intervention had good outcomes as a whole. |
| Kokai M, et al. 2004 | Japan  Philippines  Other Asian countries | Miyakejima Unzen  Pinatubo  No other volcanoes were specified | Erupting volcano | Volcanic activity in general | 1993  1991  No other years were specified | General population | Narrative literature review | Articles reviewed show that disaster psychiatry in Asia is beginning to emerge from and leave behind the stigma attached to mental health. The emergence of the acceptance of disaster mental health throughout Asia can be attributed in part to the acceptance of the notion of PTSD. This has allowed greater involvement of mental health professionals in providing ongoing support to survivors of natural disasters as well as providing greater opportunities for further research. Also, articles reviewed in the present paper commonly suggested the need for using standardized diagnostic tools for PTSD to appropriately interpret the discrepancy of results among studies. The importance of post-disaster support services and cultural differences is highlighted. |
| Takagi Y, et al. 2021 | Japan | Ontake | Erupting volcano | Volcanic activity in general (ash exposure was partially mentioned) | 2014 | General population | Epidemiological study | The affected population characteristics, victim attributes, severity of damage sustained, and evacuation status were the chief factors that influenced acute-stage mental health symptoms. The psychiatric symptoms detected in our study together with the results of diagnoses are important for determining the types of early interventions needed during the acute stage of a disaster. By sharing baseline mental health information, together with disaster-related characteristics highlighted in this study, mental health providers are better able to predict future possible mental disorders and symptoms. |
| Warsini S, et al. 2015 | Indonesia | Merapi | Erupting volcano | Volcanic activity in general (ash exposure was partially mentioned) | 2010 | General population | Epidemiological study | Two years after the eruption, survivors from the area closest to the eruption had significantly higher Impact of Event Scale Revised scores than those in the comparison area. In particular, females, adults between the ages of 18 and 59, and people who owned their own home experienced the highest levels of psychosocial impact. |
| Lima B, et al. 1989 | Colombia | Nevado del Ruíz | Erupting volcano | Volcanic activity in general (ash, pyroclastic and mud flows were mentioned) | 1985 | General population | Book | Seventy-eight percent of all the deaths occurred in developing countries, where 97.5% of the affected people were located. The observed ratio between affected and killed is only 2.9 for developed nations, but ten times greater in developing countries. Hence, not only are disasters more frequent in the Third World but they are also responsible for a much greater proportion of victims who, if they survive the disaster, need long-term management of their biopsychosocial needs. |
| Lima B, et al. 1991 | Colombia | Nevado del Ruiz | Erupting volcano | Volcanic activity in general (ash, pyroclastic and mud flows were mentioned) | 1985 | General population | Epidemiological study | The most frequent diagnoses were posttraumatic stress disorder and major depression. These findings indicate that a simple screening instrument can be reliably used for the detection of significant emotional problems among disaster victims. They also show that these victims are not merely distressed; rather, they present clear and treatable psychiatric disorders that center on anxiety and depression. |
| Gissurardóttir Ó, et al. 2019 | Iceland | Eyjafjallajökull | Erupting volcano | Ash (not reported) | 2010 | General population | Epidemiological study | Replies were received from 1146 participants in the exposed group (71%) and 510 participants in the non-exposed group (73%). Compared to the non-exposed group, participants living in the high-exposed area were at increased risk of experiencing mental distress GHQ-12 (General Health Questionnaire) 6-9 months following the eruption (OR 1.45%; 95% CI: 1.11-1.90). High-exposed participants were furthermore at increased risk of experiencing symptoms of PTSD compared to those living in the low-exposed area (OR 3.71; 95% CI: 1.34-15.41). |
| Shore JH, et al. 1986 | United States | St. Helens | Erupting volcano | Volcanic activity in general (ash exposure was partially mentioned) | 1980  Continuous activity (until 2008) | General population | Epidemiological study | These Mt. St. Helens disorders included depression, generalized anxiety, and posttraumatic stress reaction. There was a progressive “dose-response” relationship in the comparison of control, low-exposure, and high-exposure groups. The dose-response pattern occurred among both the bereaved and the property-loss victims. |
| Goto T, et al. 2006 | Japan | Miyakejima | Erupting volcano | Volcanic activity in general (ash, pyroclastic and mud flows were mentioned) | 2000 | General population | Epidemiological study | Findings indicated that material loss and uncertainty of losses were significantly associated with higher rates of reported PTSD and depression symptoms. Those who relocated multiple times reported significantly higher rates of PTSD symptoms than those who relocated less frequently. Demographic characteristics such as being older, widowed, lower SES, less education, and longer length of residency on the island were also strongly associated with higher reported PTSD symptoms. Depression symptoms were strongly associated with being widowed, lower SES, longer length of residency on the island, and previous experiences of evacuations due to disasters. |
| Syapitri H, et al. 2020 | Indonesia | Sinabung | Erupting volcano | Volcanic activity in general (ash was partially mentioned) | 2010 | General population | Epidemiological study | The impacts of this eruption are physical/health impacts (cough, shortness, flu and fever), psychological impacts (trauma, anxiety and panic), social/economic impacts (crop failure and job loss), and infrastructure impacts (damaged houses, damaged roads and clean water crisis). Post-eruption adaptation strategies are from the aspects of health (medical treatment, traditional medicine mix), social/economic aspects (carrying out community activities, cultivating land and expecting food and land assistance from donors), infrastructure aspects (building huts, repairing houses, clean water treatment, and expecting operational assistance from the government). |
| Shore JH, et al. 1986 | United States | St. Helens | Erupting volcano | Volcanic activity in general (ash exposure was partially mentioned) | 1980  Continuous activity (until 2008) | General population | Epidemiological study | This psychiatric epidemiology study following the Mt. St. Helens volcanic disaster revealed a significant morbidity for psychiatric disorders. The increased prevalence showed a dose response pattern in three population groups. The findings are reported as relative and attributable risk for the two exposed populations as compared to a control group. Patterns of significant risk are presented for sex, age, and for victims with pre-existing physical illness. |
| Ohta Y, et al. 2003 | Japan | Unzen | Erupting volcano | Volcanic activity in general (ash, pyroclastic and mud flows were mentioned) | 1991-5 | General population | Epidemiological study | Psychological distress in 248 evacuees from a volcanic eruption was evaluated using GHQ-30 (General Health Questionnaire) at four time points after evacuation: 6 months, 12 months, 24 months and 44 months. The proportion of evacuees with psychological distress (defined as a GHQ score >/= 8) significantly decreased from 66.1% (6 months) to 45.6% (44 months). The GHQ mean score significantly improved from 12.6 to 8.9. Investigation of each factor on the GHQ showed progressive improvement over time in 'anxiety, tension and insomnia' and 'anergia and social dysfunction'. However, 'depression' began to improve only after 44 months and 'interpersonal dysfunction' started to worsen after 12 months. |
| Warsini S, et al. 2014 | Indonesia | Merapi | Erupting volcano | Volcanic activity in general (ash was partially mentioned) | 2010 | General population | Epidemiological study | The I-EDS was used to collect data. Exploratory statistical methods and multivariate linear regression analyses were performed to examine the relative contributions of demographic variables on the psychosocial impact of living in an environment damaged by volcanic eruption. A total of 348 survivors of the Mt Merapi eruption participated in the survey. The mean I-EDS score for Cangkringan district was 15.8 compared to 14.6 (SD 1.3; range 11.8-18.3) for Pakem district (p<0.001). This result was confirmed by multiple linear regression analysis showing further that older respondents (p<0.001), unemployed and retired respondents (p=0.007), and respondents with no formal school education (p=0.037) had lower I-EDS scores compared to the respective reference groups. |
| Vengoechea J, et al. 1987 | Colombia | Nevado del Ruiz | Erupting volcano | Lahars | 1985 | General population | Epidemiological study | The fundamental defense mechanism was denial (98.3%), which then gave way to somatization linked to wounds and other important mechanisms. Likewise, feelings of guilt had a very important meaning. |
| Lima B, et al. 1987 | Colombia | Nevado del Ruiz | Erupting volcano | Volcanic activity in general (ash, pyroclastic and mud flows were mentioned) | 1985 | General population | Epidemiological study | Fifty-five percent of the victims were found to be emotionally distressed. Variables associated with the presence of emotional distress included living alone, having lost a previous job, feeling not being helped, not knowing the date for leaving temporary shelter, being dissatisfied with living arrangements, complaining of non-specific physical symptoms or epigastric pain, and presenting several physical problems. The high prevalence of emotional distress supports the need to deliver mental care to disaster victims in developing countries through the primary level of care. |
| Muir JA, et al. 2019 | Indonesia | Merapi | Erupting volcano | Volcanic activity in general | 2010 | General population | Epidemiological study | The results suggest that while moving home was an improvement from being displaced, it may have been better to move on, as this yielded superior associations with self-reported mental health. |

**Abbreviations:** *CI*: Confidence interval; *GHQ*: general health questionnaire; *I-EDS*: Indonesian-environmental distress scale; *PTSD*: post-traumatic stress disorder, *OR*: odds ratio. †: included by PICO of cardiovascular diseases; ¥: included by PICO of respiratory diseases; ‡: included by PICO of ocular diseases.

### Abbreviations:

- AIHDs: acute ischemic heart diseases
- AMI: acute myocardial infarction
- As: arsenic
- ATPD: acute and transient psychotic disorder
- CIHDs: chronic ischemic heart diseases
- Cd: cadmium
- CIHDs: chronic ischemic heart diseases
- Cl_2_: diatomic chlorine
- CO: carbon monoxide
- CO_2_: carbon dioxide
- COPD: chronic obstructive pulmonary disease
- CO_x_: carbon oxides
- CRDs: chronic respiratory diseases
- COVID-19: coronavirus disease 2019
- CVDs: cardiovascular diseases
- D/A: during or after
- DNA: deoxyribonucleic acid
- dysfunc.: dysfunction
- FEV_1_/FVC: forced expiratory volume in 1 second/forced vital capacity ratio
- FEV_1_: forced expiratory volume in 1 second
- FVC: forced vital capacity
- H_2_S: hydrogen Sulfide
- H_2_O: hot water vapor
- H_2_SO_4_: sulfuric acid
- HCl: hydrochloric acid
- HF: hydrofluoric acid
- HNO_3_: nitric acid
- H_x_SO_x_: hydrogen sulfide oxides
- ICU: intensive care unit
- IgE: immunoglobulin E
- IL: interleukin
- inmunodysfunc.: inmunodysfunction
- IVHHN: International Volcanic Health Hazard Network
- LTs: leukotrienes
- MADD: mixed anxiety and depression disorder
- MeSH: Medical Subject Headings
- N-FAs: non-food allergies
- NO_2_: nitrogen dioxide
- NO_x_: nitrogen oxides
- OR: odds ratio
- PAF: platelet-activating factor
- Pb: lead
- PDD: persistent delusional disorder
- PGs: prostaglandins
- PGs: prostaglandins
- PICO: population, intervention, comparison, outcome
- PM: particulate matter
- PRISMA-ScR: preferred reporting items for systematic reviews and meta-analysis extension for scoping reviews
- PTSD: post-traumatic stress disorder
- ²²²R: radon
- ROS: reactive oxygen species
- SO_2_: sulfur dioxide
- S₈: elemental sulfur
- SOB: shortness of breath
- SO_x_: sulfur oxides
- TBSA: total body surface area
- Th cell: T helper cell
- TNF-a: tumor necrosis factor alpha
- UVR: ultraviolet radiation
- Vog: volcanic smog
- µm: micrometers
